# Portfolio Decision Analysis Framework for Value-Focused Ecosystem Management

**DOI:** 10.1371/journal.pone.0065056

**Published:** 2013-06-18

**Authors:** Matteo Convertino, L. James Valverde

**Affiliations:** 1 Department of Agricultural and Biological Engineering—Institute of Food and Agricultural Sciences, University of Florida, Gainesville, Florida, United States of America; 2 Florida Climate Institute, University of Florida, Gainesville, Florida, United States of America; 3 US Army Engineer Research and Development Center, US Army Corps of Engineers, HQ, Washington, DC, United States of America; National Institute of Water & Atmospheric Research, New Zealand

## Abstract

Management of natural resources in coastal ecosystems is a complex process that is made more challenging by the need for stakeholders to confront the prospect of sea level rise and a host of other environmental stressors. This situation is especially true for coastal military installations, where resource managers need to balance conflicting objectives of environmental conservation against military mission. The development of restoration plans will necessitate incorporating stakeholder preferences, and will, moreover, require compliance with applicable federal/state laws and regulations. To promote the efficient allocation of scarce resources in space and time, we develop a portfolio decision analytic (PDA) framework that integrates models yielding policy-dependent predictions for changes in land cover and species metapopulations in response to restoration plans, under different climate change scenarios. In a manner that is somewhat analogous to financial portfolios, infrastructure and natural resources are classified as human and natural assets requiring management. The predictions serve as inputs to a Multi Criteria Decision Analysis model (MCDA) that is used to measure the benefits of restoration plans, as well as to construct Pareto frontiers that represent optimal portfolio allocations of restoration actions and resources. Optimal plans allow managers to maintain or increase asset values by contrasting the overall degradation of the habitat and possible increased risk of species decline against the benefits of mission success. The optimal combination of restoration actions that emerge from the PDA framework allows decision-makers to achieve higher environmental benefits, with equal or lower costs, than those achievable by adopting the myopic prescriptions of the MCDA model. The analytic framework presented here is generalizable for the selection of optimal management plans in any ecosystem where human use of the environment conflicts with the needs of threatened and endangered species. The PDA approach demonstrates the advantages of integrated, top-down management, versus bottom-up management approaches.

## Introduction

**Figure 1:**
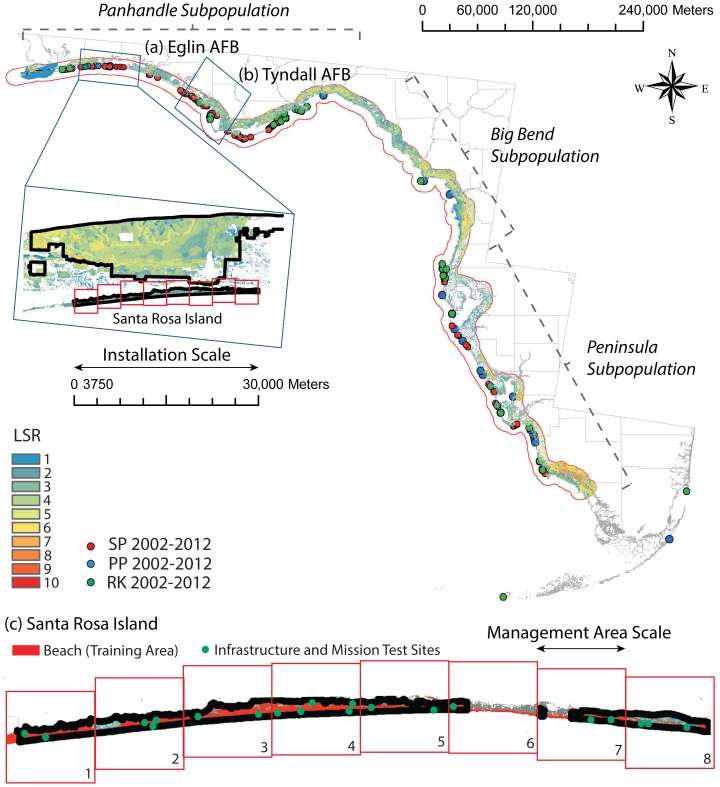
Region of the case study. The local species richness (LSR) and the occurrence of Snowy Plover (SP), Piping Plover (PP), and Red Knot (RK) are reported in the map. The Panhandle – Big Bend – Peninsula within the black line is the region considered in our biophysical modeling effort (land cover, habitat suitability, and metapopulation model). Considering the extent of each management area (3750 m^2^), 192 management areas in the whole Gulf of Mexico coastal ecosystem of Florida were considered. Eight management areas cover the portion of Santa Rosa Island managed by Eglin Air Force Base (EAFB) (a). (b) Tyndall Air Force Base that is the hotspot of SP, hosting about 60% of the whole SP population in Florida.

Military installations worldwide often harbor threatened and endangered species (TERs) that utilize habitat undisturbed by human activity [1]–[6]. The dual goal of sustaining military activities and protecting TERs in the face of changes in land cover/use and increases in human population, coupled with potential sea level rise and increases in storm frequency/intensity create significant planning challenges for natural resource managers [1], [2], [7]. For example, Santa Rosa Island (SRI), managed by Eglin Air Force Base (EAFB) in Florida, requires sandy beaches for training; concomitantly, the same habitat is utilized by the Snowy Plover, an endangered resident species in Florida (Figure 1, S1 and S2 in File S1) [2], [8], [9], and by other migrant shorebirds like the Piping Plover and the Red Knot (Figure 1 and Figure S1 in File S1) [2]–[6], [10]–[12]. Of course, EAFB also maintains infrastructure of importance for humans (e.g., military buildings, training lands, and recreational facilities, etc.; Figure S2 in File S1), as well as habitat features of importance for ecological receptors (e.g., ephemeral pools, dunes, and breeding areas) (Figure 1) [8], [9], [13]. Decision contexts such as this present environmental managers with challenges in selecting appropriate management actions that meet competing objectives pertaining to mission needs and requirements, on the one hand, and environmental preservation, on the other.

Environmental management decisions are often suboptimal, made solely on the basis of biophysical models that assess habitat needs of TERs, together with scenarios of TERs' habitats [2], [14], [15]. At SRI, Snowy Plover (SP), Piping Plover (PP), and Red Knot (RK) are the species of concern, since they are using beach and coastal marsh habitats that are threatened by the effects of sea level rise on the Gulf of Mexico [2]. Biophysical models based on Geographic Information Systems (GIS) indicate that climate change induced sea level rise negatively affects habitat area and suitability necessary for sustaining these TERs and other coastal species [2]. Evidence suggests that both habitat area and habitat suitability may be declining in the future [2]. In a similar vein, the types of areas necessary for military activities (e.g., training area, munitions testing, etc.; Figure S2 in File S1) may also be declining. Even though biophysical models can be used to identify specific areas where habitats are most vulnerable/sensitive to stress, used in isolation, such models do not provide a suitable framework for addressing specific courses of action in space and time [2], [7]. Moreover, these models do not provide decision-makers with a rational basis for comparing management actions that may benefit one species or damage another, considering, also, factors such as cost and relevant stakeholder preferences [14] Along the coast of Florida, military training, recreational use, and species conservation are conflicting objectives (Figure S2 in File S1), in that these activities compete for utilization of the same habitat. In consequence, considerable need exists for quantitatively rigorous management tools that provide prescriptive guidance to decision-makers, integrating environmental, social, and economical factors within a holistic, risk-based framework.

### Previous Approaches

GIS and multi-criteria decision analysis [16] have, individually, been used to address the needs of environmental managers. Taken in isolation, both approaches have limitations. GIS models, for example, are traditionally used to visualize data from biophysical models, and are sometimes used to solve spatial optimization problems [17]. In this vein, GIS models are well-suited to mapping important habitat features, and to finding areas where certain combinations of these features may result in increased vulnerability [17], [18]. These benefits notwithstanding, GIS-based approaches rarely consider cost as a constraint in the selection of “best” intervention sites. In those few instances where costs are considered, it is usually included as an explanatory variable that may, in some cases, yield predictive power to the overall modeling effort. One such example is the South Florida Ecosystem Portfolio Model [19], which considers cost as one of its spatial variables, in conjunction with several other relevant environmental variables.

In contrast, MCDA models provide a framework for prioritizing restoration actions based on—among other things—a hierarchy of objectives and criteria, each weighted or assigned a measure of relative importance, on the basis of value judgments and/or technical relevance to stated goals [17], [18], [20], [21]. These approaches generally focus on prioritization/ranking-related decision contexts (at predefined scales), with the primary emphasis placed on selecting *one* alternative for *one species*, rather than on *multiple*, *co-occurring* alternatives, for *multiple species*. Even though there have been attempts to integrate GIS and MCDA, most of these efforts lack spatial explicitness [20], [21]. In particular, the GIS-MCDA integration attempts reported in the literature lack the ability to integrate spatially conflicting needs; as we discuss below, this is an important requirement in multiple species management at military installations, where decision-makers must often face the difficult task of having to reconcile competing mission and conservation objectives.

**Figure 2:**
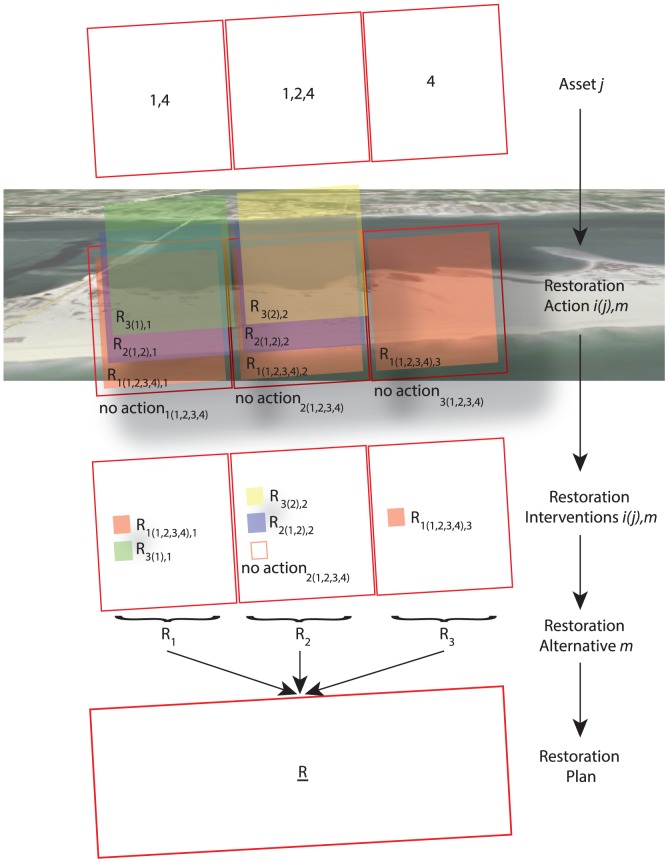
Management scales and actions. An example is reported for three management areas in SRI. Assets in each management area are indicated by the number contained in each area (e.g., 1 is the SP, 2 is the PP and 4 is the military area). Thus, management area one has two types of assets, management area two has three types of assets, and management area three has one type of assets. At least one action for each asset is evaluated in each management area. The restoration actions are indicated as *R_i(j),m_* (where, *i* is the action, *j* is the asset, and *m* is the management area). Same actions can be evaluated for different assets (e.g. nourishment) because different assets can benefit from the same actions. Only one action is selected by the PDM or by the MCDA model. The selected actions are called restoration interventions. The whole set of restoration interventions in a management area is called restoration alternative, and the set of restoration alternatives is defined as restoration plan.

A key theme advanced here is that environmental managers should look for *multiple actions* that can be simultaneously implemented at the managed site, or at different spatial units within the site, to increase overall restoration benefits and to reduce total costs. Typically, the selection of these actions focuses on maximizing *local* benefits within each site or management unit, rather than on maximizing *global* benefits; this ends up creating a “tragedy of the commons” dilemma, where the reciprocal influence or synergistic effects of restoration actions are not considered [81]. Modern portfolio decision analysis (PDA) [22]–[26] provides a set of conceptual and methodological frameworks for selecting a *portfolios* (i.e., combinations) of actions in space and time [27]–[29] (Figure 2). Even though applications of portfolio methods to environmental management problems are relatively rare, several papers consider natural resources (such as specific species of interest and habitat types) and infrastructure as “assets” that have beneficial value over time [69]. Human and natural assets can, in this way, be characterized in a manner that is, in somewhat analogous to financial assets [25], [26], [30]–[39]. Portfolio methods have also been applied to water resource management problems [40], [42], the design of natural and human systems [41]–[81], [82], [83], forestry [74], agriculture [75], fishery [76], and land use [77]; still, applications focused on the design and management of large-scale ecosystems is an important gap in the extant literature.

### Proposed Approach

**Figure 3:**
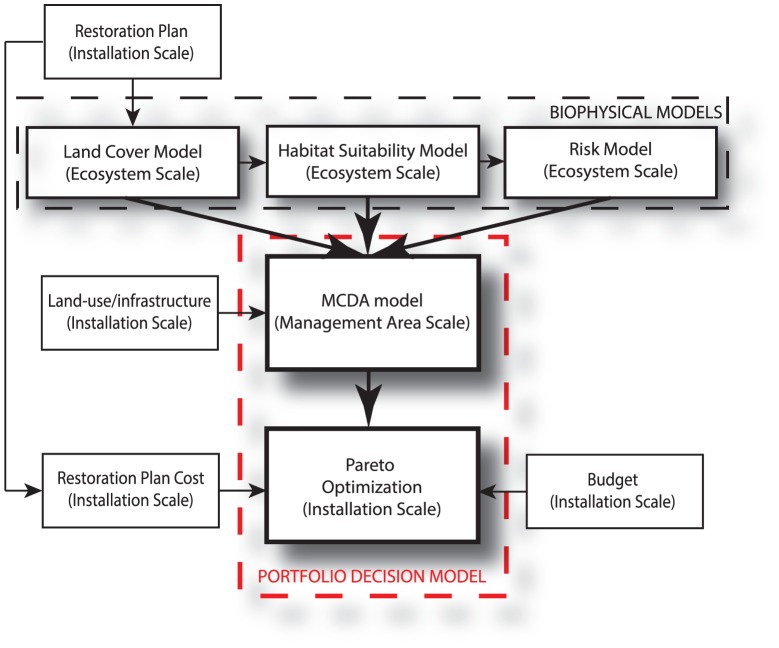
Diagram of the modeling framework. The Multi Criteria Decision Analysis model (MCDA) evaluates restoration actions for each human and natural asset at the management area scale. Outputs of biophysical models at 120 m resolution are averaged at the management area scale (3750 m). These models are run at the whole ecosystem scale that is the population scale (Figure 1) if assets are species. These outputs are part of the criteria in the MCDA model. The risk model modifies the MCDA values by considering the vulnerability of the restoration plan for each asset at the ecosystem scale and the effectiveness of each action at the management area scale (Eq. 2). The expected values of restoration actions are the inputs of a Pareto optimization model together with the cost of the restoration plan and the constraint of the budget at the installation scale. The Pareto optimization provides Pareto frontiers of optimal restoration plans at each year in the management horizon (Figure 4).

Here we present an integrated modeling approach for the selection of optimal portfolios of management actions (Figure 2). The model's decision basis focuses on the conservation of habitat for military mission and (possibly) recreational use, together with the preservation of species of concern within ecosystems in military installations. At the center of our framework is a portfolio decision model (PDM) that integrates GIS-based model predictions via an MCDA model, together with a Pareto optimization framework that looks to maximize overall (risk-adjusted) value, subject to several relevant constraints (Figure 3).

The PDM adopts several foundational concepts drawn from modern portfolio theory (MPT), none more important, however, than the concept of Pareto optimality. Whereas MPT focuses primarily on the task of developing computationally tractable means by which to allocate wealth among various expenditures and investments over time, our primary focus here is on developing a holistic, top-down approach to environmental management, with the integration of detailed biophysical models. In this paper, the value of each asset is not something that is exogenously specified or modeled (as is often the case in MPT models), but rather a spatially explicit variable that depends on a set of criteria that evolve dynamically over time, as a function of sea-level rise and restoration actions. These assets share the same habitat; however, their level of interaction is, on average, low [1]–[10]. For example, Snowy Plover, Piping Plover, and Red Knot populations are weakly correlated in term of abundance. This is due to their different spatial and temporal distribution within the same habitat [1]–[10]. Correlation between military installations and species is considered endogenously within the biophysical models employed [1]–[10]; in consequence, no further correlation is needed for the purposes of the portfolio analysis conducted here. Lastly, the correlation among restoration actions, for the sets of actions considered here, is also very small or negligible (for instance, monitoring of species does not influence nourishment).

In the case of SRI, the Pareto optimization yields, among other things, Pareto frontiers that represent the optimal combination of restoration actions that maximize the global value of human and natural assets for the military installation. Each point on the Pareto frontier corresponds to a restoration plan for the whole domain considered. Solutions that are some distance removed from the frontier may result in gains to one asset that may be offset by losses in another [21]; in the constrained case, budget (and perhaps other) constraints may restrict the ability to achieve higher global benefits than those contained within a given Pareto optimal set.

A key novelty of the PDA approach is its risk-based evaluation of local restoration actions, for multiple assets at the management area scale, as a function of their possible spatial combination at the installation scale. The best combination of actions is taken at the installation scale, with an objective function that maximizes the expected global value of all feasible restoration plans that appreciably affect the installation's assets. Such an approach is in contrast to “bottom-up” approaches that select the best action in each management unit, or to *ad hoc* “top-down” approaches that select just a few actions applied at the installation scale, without searching the relevant combinatoric space and without considering the reciprocal influence of these actions on all assets. The PDM integrates the results of GIS-based biophysical models [1], [2], [52], [53], upscaled to the management area scale via a spatially explicit Multi Criteria Decision Analysis model (MCDA) that considers relevant stakeholder knowledge and preferences. In this way, the PDM integrates and evaluates three distinct pieces of information: (i) a precise biophysical representation of human-influenced natural dynamics at the sublocal scale; (ii) the scale of heterogeneities for local scale management; and (iii) decisions evaluated at a requisite scale for decision-makers to effectively manage the system (i.e., the installation scale). In this manner, the PDM enables the simultaneous consideration of environmental, social, and economic factors in arriving at sustainable and optimal designs for environmental management restoration plans.

## Materials

### Portfolio Management Framework and Case-Study

The assets considered for Santa Rosa Island (SRI) include military areas (e.g., physical infrastructure, together with military training/testing areas shown in Figure S2 in File S1), as well as selected threatened and endangered species (e.g., Snowy Plover, Piping Plover, and Red Knot) (Figure 1 and S1). For our purposes here, assets are indexed by the subscript *j*. Other species, such as recreational and private infrastructure, occur in SRI (SI Materials and Methods); here, we focus only on assets that can be managed by the decision-maker (i.e., EAFB) and on selected threatened and endangered species that are appreciably impacted by habitat human use and by sea level rise. Military areas are also affected by sea level rise.

A subset of the management problem is shown in Figure 2, and the portfolio framework is shown in Figure 3. Installation managers can perform *restoration actions*, *r_i_(j)*, which are the types of actions feasible to restore asset type *j* (SI Materials and Methods). Restoration actions considered in the case study include beach nourishment, restoration of vegetation, predator management, limitation of recreational use, monitoring, and “No Action” (Tables S1–S4 in File S1) [52]. Restorations actions are indexed by the subscript *i* (Figure 2) The dimensionality of *r_i_(j)* can vary with *j*, and *H_j_* denotes the maximum value of *i* for asset type *j*. *Management areas* are the areas within SRI where restoration actions can be performed (Figure 2). Management areas are indexed by the subscript *m = 1, …, M*. The extent of management areas is determined by evaluating the trade-off between the need to locally capture the assets and the scale at which the restoration is feasible (SI Materials and Methods). Management areas can be also defined in terms of locations and areas where installation commanders wish to consider implementing restoration actions deemed potentially important for supporting military missions and/or for sustaining the natural environment.

We define *restoration interventions* as the restoration actions selected by the PDM or the MCDA model from the set of delineated restoration actions (Figure 2). A restoration intervention *R_i(j),m_ = 1* if *r_i_(j)* is the restoration action selected in management area *m* for asset *j*, and *R_i(j),m_ = 0* otherwise. Accordingly, the set of possible restoration actions is denoted as a vector *R_(j),m_ = R_1(j),m_, …, R_Hj(j),m_*. The choice of intervention is, for our purposes here, limited to one restoration action per area per asset. *Restoration alternatives*, *R_m_*, denote the set of restoration interventions for all assets in management area *m* (Figure 2). *R_m_* denotes the jagged array *[R_(1),m_, …, R_(j),m_]*. In the case study, restoration actions for different assets are not mutually exclusive within the same management area. Thus, different restoration actions can be part of the same restoration alternative, without negative feedbacks among each other. If an identical restoration intervention is selected for different assets, a special decision rule is involved to keep just one intervention is kept in the global optimization solution set. Nevertheless, the potential benefit of the restoration intervention is, nevertheless, considered for both assets.

*Restoration plans*, *R*, are defined as the ensemble of all the restoration interventions for every management area (Figure 2). Equivalently, a restoration plan can be defined as the ensemble of all the restoration alternatives in the ecosystem analyzed. For our purposes here, we evaluate the performance of restoration plans selected by using the MCDA model and the PDM (Methods). The *cost* associated with each restoration action is a relative value estimated from the literature, and is denoted by *C_i,j_*. The cost of a restoration action is assumed to be independent of the location. The cost of a restoration plan, *C_T_*, is simply the sum of all selected restoration interventions in SRI by the MCDA or the PDM.

Let *V_m,j_(R)* denote the *local value* for restoration plan *R* with respect to asset *j* in management area *m* (Eq. 1) The local values of assets are calculated using the MCDA model based on criteria values and asset criteria weights (Methods, and examples of Tables S5 and S6 in File S1). *Criteria values* are the MCDA-derived values for an asset, as a function of restoration plan *R* in a management area *m* (Methods). Criteria values, and thus local values, change each year in the simulated period, as a function of the sea level rise scenario of 2 m (A1B) (SI Materials and Methods). A criteria value *x_j,k,m_(R)*, *0≤x≤1*, defined on the unit interval, is the value for asset type *j* on criterion *k* in management area *m* under restoration plan *R*. *Asset criteria weights*, *w_j,k_*, are used to measure the relative importance of each criterion *k* to the overall performance of asset *j*. The sum of all weights for an asset are normalized and sum to unity. In this way, assets can be valued differently by stakeholders, and may play roles of differing importance for sustaining ecological receptors; intuitively, some of them may be more important than others for achieving specific goals. Accordingly, we introduce *stakeholder asset weights*, *w_j_*, that define the relative preference of different assets for stakeholders. We define stakeholders as all the individuals (experts, laypersons, decision-makers, organizations, etc.) with a stake in the decision problem considered (Wood et al., 2012). These weights may also reflect different levels of prioritization for specific assets, due to legal and/or economic constraints. As before, the sum of all stakeholder asset weights is normalized so that they sum to one.

Restoration actions change the value of assets in management areas where these assets occur. In this way, restoration actions affect criteria that characterize assets. In this way, the variation of criteria expresses the variation of benefits determined by restoration actions. The local values of assets are modified by the vulnerability of the assets calculated by the risk model given the restoration plan globally and the effectiveness of the restoration action locally (Figure 3). Ideally, the calculated MCDA values should represent the holistic values of assets, considering both local and global management. For this purpose, we introduce an *effectiveness factor*, *f_i(j)_*, that represents the overall capability of restoration action *r_i(j)_* to increase the value of asset *j*. The effectiveness coefficient is defined for all restoration actions (Tables S1–S4 in File S1) [54]–[63], except for the nourishment and “no-action” options. The nourishment and no-action policies have been simulated using biophysical models (SI Materials and Methods), thus their criteria values endogenously model the effectiveness of these actions. We define *f_j_* as the vector f_1(j)_, …, f_Hj(j)_ that multiplies the MCDA value of the no-action restoration to obtain the MCDA value of the other restoration actions considered here. The effectiveness of each action is assessed from the relevant literature: for the predator management [57]; for the restoration of ephemeral pools and beach profile [12], [61]; for nourishment [12], [52]; for limitation of recreational use, monitoring, and restoration of dune vegetation [61].

The *vulnerability*, *v_j_*, of asset *j* is defined as the risk of loss or decline of the asset as a function of restoration plan *R*. The risk of assets is calculated on the basis of the magnitude and frequency of sea level rise, asset vulnerability, and exposure under uncertainty and different restoration plans. In this way, vulnerability is a measure of how each restoration plan withstands changes in habitat area and quality determined by sea level rise for a particular asset. For human assets (military areas, MA hereafter), the vulnerability is defined as a function of the expected probability that the habitat area is preserved. The vulnerability is given by the complementary of the ratio between the habitat at each time step and the habitat in 2013. For natural assets (SP, PP, and RK), the vulnerability is determined as the expected decline of the species population. Such declines occur when the mortality (and emigration) rate is greater than the birth (and immigration) rate for the period considered, where the population size reaches a value lower than a critical population threshold. The population threshold is set to twenty for the SP, and RAMAS [64] is used for the metapopulation risk modeling [1]. The risk model (Figure 3) considers the whole time horizon and all the subpopulations along the Florida Gulf coast (Figure 1). For the PP and the RK, the probability of extinction/decline of species is derived from the International Union for Conservation of Nature (IUCN) index [65]–[67], which is normalized to the maximum IUCN index of the species considered. The IUCN index is 1–6/7 (Near Threatened over seven classes of conservation status) for the PP and the RK. The IUCN is an acceptable surrogate metric of the vulnerability when a metapopulation model cannot be run for the species considered [66], [67]. The IUCN Red List categories are intended to reflect the likelihood of a species going extinct under prevailing circumstances. For the human assets, vulnerability is assessed by the land cover model evaluated at the Florida Gulf coast scale.

The *expected local value*, V^*^_m,j_(R), (Eq. 2 below) of the restoration plan is calculated for each management area *m*, considering its probability of success as a function of the vulnerability of asset *j* and the local effectiveness of the restoration actions selected. The expected local risk is simply the product of the complementary of the local value, the vulnerability, and the complementary of the effectiveness coefficient. The PDM takes as input these expected local values to calculate *V_T_(R)* (Eq. 3), which is the *global value* of human-natural assets at the installation scale. *V_T_(R)* is calculated by considering all of the local expected values of human and natural assets for a given restoration plan. These local values are then weighted by stakeholder asset weights prior to entering the PDM. The global value is determined by maximizing the objective function via a Pareto optimization algorithm for determining the optimal restoration plan (Methods). The optimal restoration plan is defined as the plan that maximize the expected value of assets at the global scale, with a restoration plan cost that is less than or equal to the available budget *B*. The global value can, itself, be disaggregated into two discrete components: a global value for human assets, *V_H_(R)*, and a value for natural assets, *V_N_(R)*. In maximizing the objective function, it is therefore possible to choose Pareto optimal restoration plans that favor human and/or natural assets, with different relative importance values specified at the installation scale.

For ease of analysis and exposition, we make a number of simplifying assumptions in numerically specifying the proposed portfolio model for ecosystem management. We assume, for example, that the cost of restoration actions is invariant in space and time. In addition, no discount rate has been incorporated for either budget and restoration action costs (though, clearly, in extending the model presented here to dynamic decision contexts, such an assumption would be untenable, especially in situations characterized by long planning horizons). We further assume that there is no species adaptation to climate change in the time horizon considered. Habitat requirements and metapopulation parameters are therefore constant in time. All of these assumptions can be modified to suit particular instances, applications, and purposes. Our goal here is to outline the core elements of a spatio-temporal portfolio decision model that aggregates together environmental models, decision models, stakeholder preferences, and economic constraints at the management scale. In doing so, we consider the heterogeneity at smaller scales in the distribution of assets, the needs of biophysical models, and the scale of decisions dictated by the scale at which decision makers can implement management decisions.

## Results

The effects of Sea Level Rise (SLR) on the habitat, e.g., by diminishing beach areas [5], [6] and habitat suitability [2], increase the need for major restorations in time (Figures S3, S4, and S5 in File S1). The value of the criteria maximized within the MCDA model (e.g., habitat area and suitability) decreases as sea level rise increases on average (Figure S3, Tables S5 and S6 in File S1). The habitat suitability of the Snowy Plover [68] for different years (2013, 2060, and 2100) and scales (whole coast and Eglin AFB, respectively) is shown in Figures S4 and S5. The habitat area and suitability rapidly decreases after 2060, at which point SLR begins to rapidly increase to 2 m in 2100 (Figure S3 in File S1). The change in value of criteria that are minimized in the MCDA model due to SLR, and specifically the abundance fluctuation and the habitat fragmentation, is different depending on the particular criterion and management area considered. For this reason, the MCDA value of a restoration action fluctuates around an average value, and it is difficult to conclude if the local value of assets increases or decreases as a function of SLR in a given management area. The global value for all restoration interventions of all assets at the SRI scale decreases on average as sea level rise increases. This is observed in the “No Action” scenario. The MCDA values are used for selecting restoration plans, with both the MCDA-based and the PDM-based selection methods.

### Portfolio Decision Model Selection

**Figure 4:**
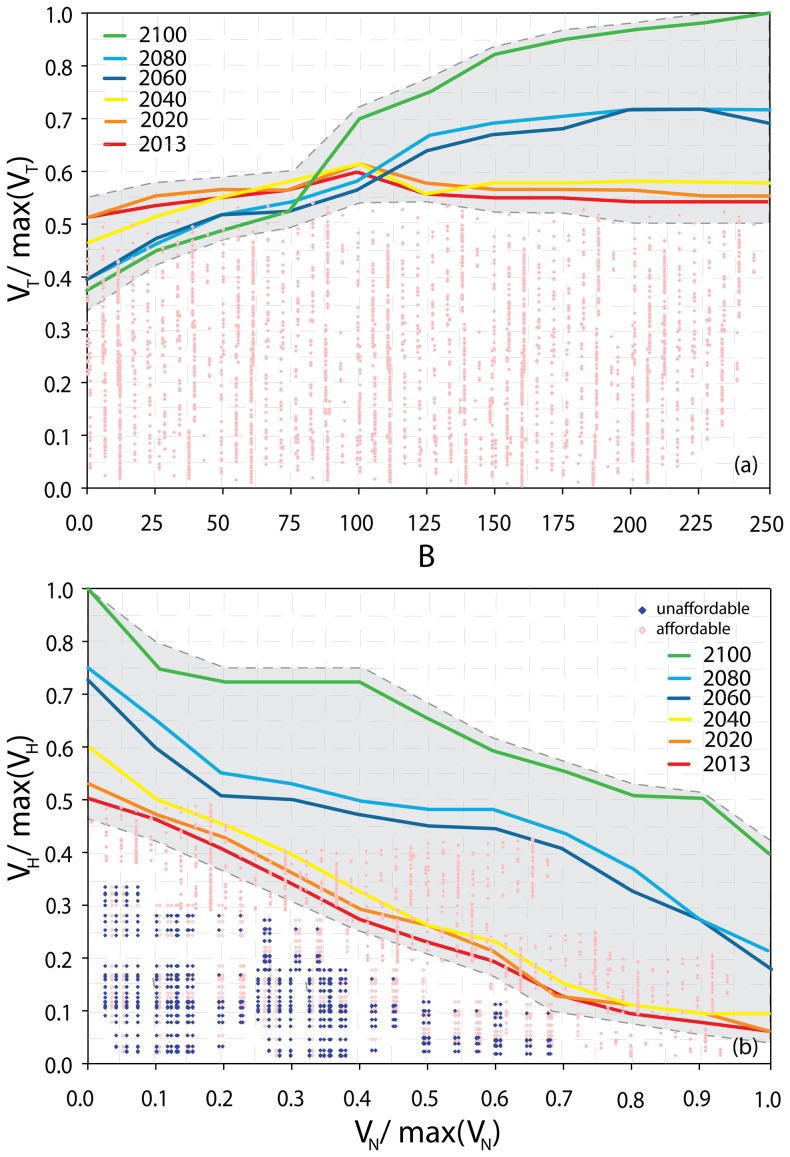
Pareto frontiers after realization of the selected restoration plan. (a) Pareto optimization unconstrained to the available resources. Red points represent the suboptimal restoration plans for the frontier in 2013. Grey dashed lines represent the variability of the frontiers related to thirty Monte Carlo simulations generated considering a random white noise applied to the MCDA value. (b) Pareto frontiers constrained to the resources available for selected years of analysis; we assume that the total resources available are equal to 250 at the installation scale (Eglin AFB in Figure 1). The frontiers show V_H_ and V_N_ (Eq. 2) normalized to their maximum value. The blue and red dots represent unaffordable and affordable restoration plans (or portfolios) for 2100. All the possible values of V_H_(R) and V_N_(R) for selected years are shown along the Pareto frontiers. The choice of the Pareto set depends on relative stakeholder preferences for human and natural assets.

**Figure 5:**
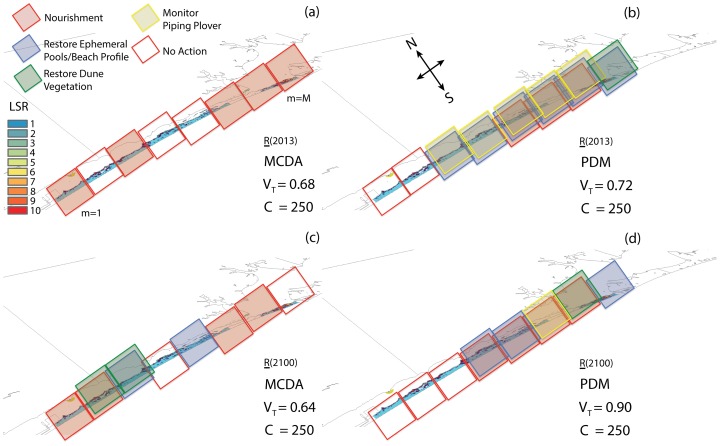
Restoration plans for Santa Rosa Island for the MCDA and PDM. Restoration plans are shown for 2013 (a, b) and 2100 (b, d) after the MCDA and portfolio decision model (a, c, and b, d respectively). The size of each management area is 3750 m^2^. The total cost of the selected restoration actions is 250 resource units that is the budget available.

The outputs of the PDM are shown in Figure 4. The optimal restoration plans for SRI selected by the PDM framework in 2013 and 2100 are shown in Figures 5b and 5d, respectively. The total number of portfolio combinations is 2^8^×5^7^×7^8^×4^1^ = 4.61×10^14^, which is simply the product of the number of possible actions for each asset (MA, PP, SP, and RK, respectively) raised to a power exponent equal to the number of management areas where each asset is present (Table S7 in File S1). Pareto frontiers for the years 2013, 2020, 2040, 2060, 2080, and 2100 unconstrained to the available budget are shown in Figure 4a. This figure shows what is known as the “productivity ratio” curve. The investment choices that lie along the surface of this curve are considered to be “efficient” in that they return the greatest possible value for any specified level of funding or resource. As one moves, then, from the left-hand portion of the Pareto frontier to the right-hand-portion, there is decreasing marginal return in value obtained for each additional increment of cost or investment.

Restoration plans, which are the individual points shown in the Pareto plot, define the set of possible restoration interventions selected for all of the natural and human assets in all management areas within SRI. Restoration plans along the Pareto frontier are optimal restoration plans, in that they maximize the global value of assets for any specified level of budget. We consider a long time horizon (2013–2100), and therefore uncertainties in sea level rise projections are an important consideration. These uncertainties are explored for each model used (SLAMM, MaxEnt, and RAMAS) in [5], [11], [79] and [6], respectively, by performing a global sensitivity and uncertainty analysis [80]. For each year, the Pareto frontier that corresponds to the maximum global value *V_T_(R)* (Eq. 3) for each budget level is generated up to a budget of 250. These forward-looking Pareto frontiers can be used together to develop a comprehensive restoration strategy for the planning horizon under consideration.

**Figure 6:**
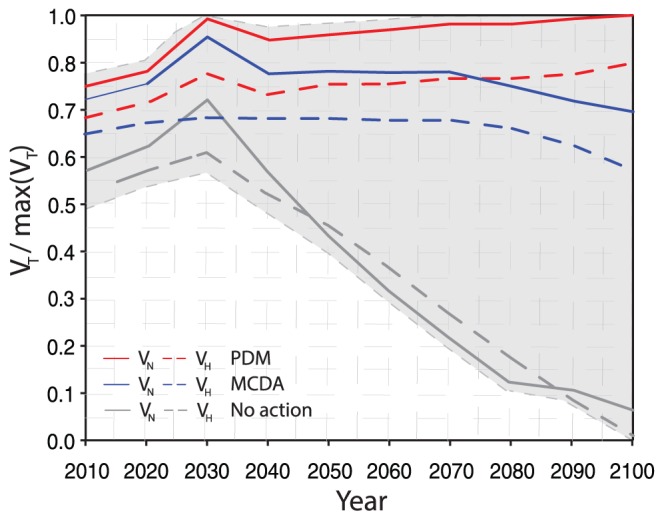
Global value disentangled into expected human and natural value of optimal restoration plans for the MCDA and PDM. The expected natural and human values (Eq. 2) are calculated by considering the vulnerability of human and natural assets given a restoration plan for the whole ecosystem and the effectiveness of each restoration intervention for each asset. The curves are shown for a budget of 250 units. We selected the Pareto set for which V_H_(R) = 0.5 V_N_(R). Grey dashed lines represent the variability of the patterns related to thirty Monte Carlo simulations generated by considering a random white noise in the MCDA value.

The Pareto frontiers in Figure 4a show that the increase in *V_T_(R)* is small for the initial years of the time horizon considered (2013–2060), for any presumed budget level. In contrast, the increase in *V_T_(R)* determined by the frontiers is higher for the years after 2060, as a function of the installation budget. These trends indicate that larger investments in restoration efforts should be potentially considered in later years after 2060, when the effects of climate change on the habitat are larger than in the initial years before 2060. The utility, then, of larger expenditure in restoration efforts should be larger from 2060 onwards. On the basis of the expected *V_T_(R)* determined by the Pareto frontiers in these periods, these numerical results suggest that the investment of resources should be approximately in the range of 0≤B≤75 from 2013 to 2060, and in the range 75≤B≤250 from 2060 to 2100. The global value of human-natural assets for a budget equal to zero shows the case where no restoration plans are adopted for any year in the time horizon considered. The global value of assets for this scenario is shown in Figure 6. For Pareto optimal plans, *V_T_(R)* in 2012–2040 is smaller than the average value for years after 2040. This observation reinforces the suggestion that large investments are necessary in later years of the planning horizon, in order to keep the expected global value at least equal to the global value in 2013. In an adaptive management context, these frontiers are updated year-to-year, without necessarily considering the relevant state history. The idea here is to provide a management tool that shows the potential global value of restoration plans if these plans are fully implemented every year, with “No Action” being taken in the previous years of the management horizon considered (from 2013 to the selected year). More realistic utilizations of the PDM would naturally consider potential trajectories of subsequent restorations plans in time, updating the Pareto frontiers of subsequent years considering the selected restoration plans in earlier years.

Because we consider the productivity ratio curves rather than the classical MPT frontiers the variance of the portfolio is represented within the grey regions. The variance is a function of the value of the criteria considered, their uncertainty, and the additional portfolio uncertainty included as truncated Gaussian noise (Methods).

The Pareto frontiers constrained to a budget of 250 are show in Figure 4b. For these frontiers, each possible restoration plan is depicted as a dot with different ratio between the global value of human and natural assets (Eq. 3). Accordingly, the frontiers show the global value, disaggregated into its respective human and natural asset values, at different years for the Pareto optimal and suboptimal solutions of Figure 4a with B = 250. The relative importance of human versus natural assets can be decided according to stakeholder preferences, or perhaps by legal constraints. In this way, the frontiers are useful for the design of restoration plans because they allow decision-makers to consider the implications of (desired or required) trade-offs among habitat-competing assets of the managed ecosystem. For instance, V_H_ can be considered as the ecosystem service of the built environment (military infrastructure), and V_N_ as the ecosystem service for the natural environment (species habitat). However, a net separation of ecosystem services does not exist, due to the overlapping nature of different land uses for assets within the same habitat. Thus, the coastal habitat is the natural resource that both natural and human assets use to perform some services, and the value of these services is captured by the natural and human values.

The tri-dimensional view of the Pareto optimal restoration plans for 2013 and 2100 (Figures 5b and 5d, respectively) simplifies the visualization of overlapping actions in management areas. Management actions are represented by squares of different colors. These plans correspond to a ratio between human and natural values that is equal to one (i.e., for V_N_/Max(V_N_) = 0.32 in Figure 4b). The PDM-based plan selection is seen to be heterogeneous in terms of restoration action types, and the portfolio restoration interventions (i.e, the actions selected) tend to be close to each other within SRI. This management heterogeneity, created by the large variety of restoration interventions, is observed for the entire time horizon.

In 2013 (Figure 5b), the nourishment selected in management areas 5, 6, and 7 (from west to east) for the military is positively affecting SP, PP, and RK that co-occur in the same management areas (Table S8 in File S1). Monitoring is the intervention that benefits just the PP from management areas 3 to 7. Moreover, the Pareto optimization includes restoration of ephemeral pools in management areas 2 to 8 (selected for the PP in area 8 and for the SP elsewhere), and the restoration of dune vegetation in management area 8. “No Action” is needed in management areas 1 and 2. The number of major restoration interventions (e.g., nourishment) is increased in 2100, as well as the number of areas where no-action is selected. In fact, the number of “No Action” options selected in Pareto optimal plans increase in time. This effect is related to both the constraint of the budget, and the need for major and costly restorations in preserving the habitat.

The frequency distribution of PDM-selected restoration interventions for SRI is shown in Figure S6 (File S1) for several relevant years in the modeled time horizon. It is noticeable that the frequency of costly restoration interventions (i.e, nourishment) increases from 2013 to 2100, and the number of “No Actions” has a peak in 2060. This trend is related to the predicted rapid increase of inundation of the coastal ecosystem starting in 2060 [5], [6], and to the very limited budget available. The available budget is, in fact, assumed to equal 10% of the total cost if all the restoration actions are performed for each asset in every management area. The constraints of the budget do not allow decision-makers to perform minor asset-specific restoration when the effect of sea level rise is major.

### MCDA Selection

The MCDA-based selection method of restoration plans occurs independently of the consideration of the budget available for the installation. Restoration actions are selected one at a time for the management areas, according to the expected MCDA value, V^*^_m,j_(R) (Eq. 2), starting from the action with the highest value within the installation. Although the selection is based on MCDA values adjusted by the vulnerability *v_j_* (Eq. 2), the selection does not consider the best *combination* of restoration actions for the installation globally. We observe that the selection is biased toward expensive restoration actions (e.g., nourishment) for any asset using the MCDA as a selection method of restoration plans (Figure 5). One reason for this is that these actions dominate other actions in terms of benefits. Table S9 (File S1) reports the interventions of the MCDA-based selection method in 2013. There, just a few less costly actions are included in the MCDA-based restoration plans (e.g., the plans in 2013 and 2100 are shown in Figures 5a and 5c, respectively). In addition, we note that the selection of actions based solely on expected MCDA values tend to select actions in pixels that are far from each other. These interventions may diminish the benefit for the assets for which they have been selected, and the benefit for assets in the rest of the habitat.

### Comparison of MCDA and PDM

A formal comparison is made of the restoration actions as prioritized utilizing the MCDA approach and those that are recommended by the PDM. The comparison is made to highlight the prescriptive implications for the two approaches. Current practice within ecosystem management tends to maximize benefits *locally*, without considering *dependencies* of these actions and their *global value* at the installation scale.

The cost and global value of restoration plans selected by MCDA and PDM can be compared. For SRI, PDM allows a higher *V_T_(R)* for the same cost in both 2013 and 2100 (Figure 5). The restoration plans in Figure 5 for 2013 are the plans that are listed in detail in Tables S8 and S9 (in File S1). The global value of plans is given by the sum of the expected local values for all the assets that benefit from the interventions of the plan. The comparison between MCDA and PDM selection methods is made by investing the available budget in its entirety. We note that the PDM plan outscores the MCDA plan with higher benefits and lower costs, even when removing some interventions from the PDM plan (see Figure 5, and Tables S8 and S9 in File S1). By considering the whole time horizon without the constraint to invest the whole budget for the PDM (“No Actions” are selected when the MCDA cannot add any other action to the plan), the PDM allows a 25% reduction in the cost of restoration plans with respect to the MCDA. The PDM plan benefits are estimated to be 20% higher than the MCDA. On average, then, the PDM with Pareto optimization results in higher benefit/cost ratios for management plans in the planning horizon. The higher benefit/cost ratio is achieved by the ability of screening any combination of restoration actions without the limitation of considering plans that just maximize locally the expected benefits of restoration actions.

The global values of assets for restoration plans selected by the MCDA model and the portfolio-based method are shown in Figure 6 for a budget of 250 units. We adopt “units” proportional to the current relative cost of restoration actions (Table S7 in in File S1). The global value is calculated for the A1B sea level rise projections through 2100 for the SRI coastal system. We assume that the Pareto optimal plan is performed each year. We also show the global value in the ““No Action”” case for every year of the analysis, management area in SRI, and elsewhere along the Florida Gulf coast. We observe that the global value of natural and human assets increases, on average, until 2065, for both the portfolio and MCDA plans. For the MCDA plan, the global value decreases from approximately 2065 to 2100; in contrast, the global value of the PDM plan continues to increase to 2100. The decrease observed in the global value of assets for the MCDA plan is related to the strong decrease in habitat area caused by sea level rise and is not replenished optimally by the MCDA restoration plan. A rapid decrease in the global value of assets is observed from 2030 for the “No Action” scenario. This result speaks to the need for restoration actions in the SRI coastal ecosystem. The value of natural assets is higher than the value of human assets because of the shape of the concave Pareto frontiers constrained to the available budget (Figure 5b). On average, V_N_ is larger than V_H_, with the exception of the “No Action” scenario, where the loss of habitat strongly affects both natural and human assets equally.

Despite the absence of explicit spatial rules in the combination of restoration actions in the PDM model (all combinations are screened), the global value of assets for restoration plans selected by the PDM is maximal for all years along the considered timeline. Restoration plans are, indeed, needed, but they should be selected by an optimization method that selects those combinations of strategies that hold the greatest potential for maximizing the global value of assets at the installation scale. The PDM is well suited to this task, as it allows the selection of the best spatial combination because the mutual location of actions and relative benefits are screened as independent variables of the managed system. In contrast, methods like traditional MCDA evaluation rank actions at the installation scale according to their local benefits, and restrict further selection of actions to plans that guarantee those local benefits. Bottom-up approaches like this erode faster the global budget at a greater rate by investing in costly actions that do not necessarily increase the value globally (many assets are not affected by restorations) and, perhaps, in the long term.

## Discussion

The proposed spatio-temporal and multiscale portfolio decision model holds the potential for being able to transform the ways in which stakeholders manage human and natural assets that are both threatened by natural stressors (such as sea level rise), and that compete with each other for the use of the same habitat. The PDM is particularly useful in ecosystems where strong trade-offs among assets appear; for example, where human uses can interfere with the preservation of natural resources, as is the case of military installations. The incorporation of multi-criteria value models allow for the systematic integration of output drawn from several biophysical models, directed at selecting optimal restoration plans. The portfolio model provides a conceptual scheme that is well-suited for the analysis of restoration plans under other climate change related stressors (e.g., changes in precipitation and temperature) and military mission scenarios. At the same time, the framework is quite flexible and robust. For example, in those cases where biophysical models are not available, expert assessments can be used as inputs. Importantly, this approach explicitly accounts for the priorities and values of stakeholders in a transparent and quantitative fashion. Resource managers can use the weighting scheme to determine how sensitive candidate restoration plans are to changes in mission objectives or parameters. The PDM approach provides a powerful analytic framework for the identification of restoration plans that consider the spatiotemporal complexity of ecosystems in response to complex and potentially conflicting needs and objectives. We believe that this approach quantitatively embraces the sustainability paradigm that brings together social, environmental, and economical factors in the effective management of ecosystems [7].

The recommendations that follow from the PDM improve upon the set of restoration interventions selected by the MCDA model alone. The Pareto optimization within the PDM allows for the identification of restoration plans that maximize value and minimize the vulnerability of assets for each year (or for a specified planning horizon) at the installation scale. By contrast, the MCDA model selects the dominant restoration actions (those single actions that have the highest MCDA value) for each asset in each management area, without considering the combinations with other restoration actions in terms of mutual benefits and costs. In this way, the MCDA model does not consider the importance of a restoration plan as spatial combination of actions at the ecosystem scale; rather, the MCDA model selects restoration actions starting from the maximization of local benefits at the management area scale. The Pareto optimal frontiers of global value of assets relative to the budget for each year are useful for the management of the ecosystem at each year in the planning horizon. In addition to the direct use of each Pareto frontier separately for each year, the set of frontiers can be used for strategically identifying feasible restoration trajectories over time, given anticipated resource availability and discount rate predictions. Ideally, with the knowledge of the resources available for restoration plans over multiple years, there are many trajectories that can be followed, moving from one Pareto frontier to another. The PDM can therefore inform simultaneous management of natural and human assets, in a manner that considers needs, costs of restorations, and stakeholder preferences.

Despite its utility, the PDM can be technically challenging. Rigorous implementation requires an investment in biophysical and decision analytic modeling. Our simplified case study requires a number of assumptions to be made, which may limit its prescriptive usefulness. Specifically, costs were assumed to be invariant and no discount rate for either costs and budget was applied. Criticality factors, the effectiveness of each action, together with costs, were estimated for each asset and restoration action, with limited data. Amending or strengthening each of these assumptions adds a level of complexity to the overall analysis, and potentially introduces new sources of error to the model. In this approach, uncertainty in the form of truncated Gaussian noise is added to the MCDA values in an effort to visualize the cone of uncertainty in the results. A better representation of the sensitivity of each model factor and their uncertainty on the selection of a restoration plan would be desirable. The incorporation of this level of investigation represents the next steps in the development of the portfolio model. The global sensitivity and uncertainty analysis will also help in determining the value of information of additional factors not yet considered and related to the assumptions made in the current version of the model.

Related research developing portfolio approaches for maximizing environmental value is limited. Reference [33] develops a method that maximizes ecological diversity, but reduces social returns. Thus, in the method of [33], maximizing biodiversity is not an optimal investment from a societal point of view. In contrast, the PDM approach developed here maximizes an objective function that simultaneously considers societal and ecological values. The objective function is the function that brings together competing and non-competing environmental and social factors, in a utility scale limited by economic constraints.

## Conclusions

Portfolio-optimized decision models should be considered in environmental management of human and natural resources. The current legal system and practice tends to adopt bottom-up management practices that maximize a few local assets. Alternatively, integrated top-down portfolio approaches like ours maximize the collective benefits of multiple assets at the management decision scale. The diversification of resources among assets of the portfolio scheme allows managers to plan and act by implementing optimal restoration plans that increase ecosystem value concomitantly with the pursuit of other goals and objectives.

## Methods

### Restoration Plans and Plan Selection

All feasible restoration plans are identified by the combination of restoration actions considered for the assets analyzed. The maximum number of possible restoration plans is given by the product of the number of alternatives to the power of the product of the number of management areas where assets occur for each asset. The value of each restoration action is evaluated by the MCDA model in each management area, and for each restoration plan. This value is then adjusted to consider the probability of success of the restoration plan for all assets. Two selection methods of restoration plans are examined: (i) a method based on the rank of the local expected value of asset; and (ii) a method based on the maximization of the global expected value of assets. The first method selects restoration actions according to the expected MCDA value, starting from the actions with the highest value until the budget B is spent. The only constraint is to select plans that observe the value of restoration actions already selected in other management areas. The second method selects the best portfolio of restoration actions that maximize the global value of assets at the global scale constrained to the budget available. Accordingly, the MCDA-based selection method tends to maximize the *local value* of each asset at the management scale; in contrast, the portfolio-based selection method maximizes the global value of all assets at the installation scale. If the same restoration action is selected for two or more assets for the same management area, the duplicated action is dropped from the restoration plan. However, the values of the duplicated restoration actions for all the assets considered are included in the formation of the global value. This is because the same restoration action benefits simultaneously multiple assets. Thus, the cumulative value aims to represent the benefits of the same intervention despite habitat competition and interactions among assets can decrease that value. In our case study, none of the restoration actions have a negative effect on other assets occurring in the same management area. The cost of only one intervention is included in the portfolio-based selection method in the case of duplicated interventions for assets in the same management area. For both approaches, the budget B is limited to 250 units for SRI. This budget is approximately 10% of the total cost if all the restoration actions are performed for each asset in every management area.

### MCDA Model

The MCDA model used for evaluating each restoration plan for human and natural assets is a linear Multi Attribute Value Theory (MAVT) model. The MCDA model ranks restoration actions by scoring them as linear combination of criteria values and criteria weights. The MCDA model calculates the local value for restoration plan *R* with respect to asset *j* in management area *m* at each of the simulation. We assume implicitly the dependence on time of the predicted values. The MCDA value that is ultimately the value of assets given a restoration plan is:
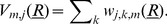
(1)The weights *w_j,k_* are assigned by stakeholders, and correspond to the relative importance of each criterion for the local value of restoration actions [16], [69]. Here, we utilize an illustrative set of weights elicited according to our expert knowledge about the problem. The MCDA model takes as input spatially explicit predictions of local- and installation-scale criteria from biophysical models [1]–[6], [10]–[12]. Each restoration action is evaluated every year for each feasible restoration plan (SLAMM, MaxEnt, RAMAS) [1]–[6], [10]–[12], [64], [70]–[72], for the planning horizon considered (2013–2100). We assume that projected sea level rise proceeds according to the A1B climate change scenario. Criteria values *x_j,k,m_* depend on the whole restoration plan R. These criteria are maximized or minimized for rescaling criteria values to increasing or decreasing value functions in a range dictated by the maximum and the minimum criteria values. For example, habitat suitability is a criterion that depends on local heterogeneities, but also on the whole configuration of the habitat along the coast. The MCDA value of assets does not take into account the likelihood of success of the restoration plan. The likely success of each restoration action is evaluated by both considering the vulnerability of each asset at the global scale and the local effectiveness of each restoration action. These factors multiply the MCDA value to obtain the expected value of assets.

### Expected Local Value of Assets

The expected local value in area *m* for asset *j* and for restoration plan *R* at each of the analysis is given by:
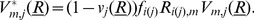
(2)The value of a restoration plan is adjusted by the probability of success given by the vulnerability of each assets at the global scale (*v_j_(R)*) and the effectiveness of a restoration plan (*f_j_ = f_i(j)_ R_i(j),m_*), considering the restoration interventions selected locally in the management areas (*R_i(j),m_*). The expected values are calculated for both the MCDA-based method and the PDM-based selection methods.

### Expected Global Value of Assets

The global value of human-natural assets, *V_T_(R)*, of a restoration plan (i.e., a set of restoration alternatives at the installation scale) is calculated as a Euclidian distance.

We selected the Euclidian distance because we hypothesize the ecosystem services as additive services. Ecosystem services are the benefits that natural and human assets can get by actions that preserve the habitat they use. The Euclidian distance allows to easily analyze the contribution of both factors. A simple sum of natural and human values produces the same Pareto frontiers bur rescaled vertically in values. The Euclidian distance is used because the space of ecosystem services is often seen as a multi-dimensional space, and thus the distance is the most proper function to consider services together.

The values of human and natural assets weighted by stakeholder preferences at the global scale, *V_H_(R)* and *V_N_(R)*, are the two components of the distance. These values are V_H_(R) = ∑_m_ ∑_j_ V^*^_H m,j_(R) w_j_ and V_N_(R) = ∑_m_ ∑_j_ V^*^_N m,j_(R) w_j_ respectively. Because of the absence of a careful stakeholder preference elicitation, in this study we assume the preferences *w_j_* to be homogeneously distributed among assets because the lack of a stakeholder preference elicitation effort. The elicitation of preferences should be performed in real-world applications of the model. The global value of a restoration plan calculated for both the MCDA-based and PDM-based selection method at each year of the analysis is given by:
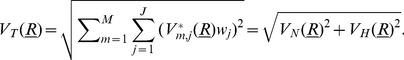
(3)


### Pareto Optimization Model

The Pareto optimization model assumes that the global value of assets for each restoration plan is a multi-objective function that maximizes (Eq. 3). We assume mutually independent restoration actions under uncertainty. The optimization is a linear mixed-integer optimization algorithm [73] that explores all possible combinations of restoration actions (for each asset, one at a time), with their expected local value and cost at the installation scale. The maximization of the global value is performed with and without the constraint of the available budget *B* of the installation, for each simulated time period. In the constrained case, the cost of the restoration plan, *C(R) = ∑_m = 1,M_ ∑_j = 1,J_ C_m,j_(R_i(j),m_)*, cannot exceed the budget *B*. In the case of the Pareto optimization unconstrained by budget, if *V_T_(R_1_)*≥*V_T_(R_2_)* and *C(R_1_)<C(R_2_)*, then the portfolio solution R_1_ dominates *R_2_*. Thus, all the restoration actions in R_1_ are selected. In the budget constrained Pareto optimization, if *V_T_(R_1_)*≥*V_T_(R_2_)* then the portfolio combination *R_1_* dominates *R_2_*.

Optimal restoration plans are defined as the Pareto-efficient solutions along the Pareto frontiers calculated by the optimization model. Pareto frontiers can also show the relative importance of natural and human assets. In our budget constrained case, we assume that natural and human assets are equally important at the installation scale. Thus, the ratio *V_H_(R)*/*V_N_(R)* is equal to one. Unaffordable plans are those for which the cost is higher than the available budget, and with global value of assets lower or higher than the Pareto optimal plans. These portfolio solutions are above the Pareto frontiers unconstrained to the budget available. Affordable plans are those whose total cost is lower than the budget, but they are suboptimal in term of global value (*C(R_1_)<C(R_2_)* and *V_T_(R_1_)*<*V_T_(R_2_)*).

The input factors of the Pareto optimization model are reported in Table S7 (in File S1). The number of restoration interventions contained in any Pareto optimal solution is equal to the product of the number of assets considered and the number of management areas including the “No Action”.

### Uncertainty Analysis

To consider uncertainty in the selection of restoration plans we add a truncated Gaussian noise term μ in the local values of assets determined by the MCDA model (Eq. 1). The noise is assumed to fall in the range [−0.05, 0.05], with mean and standard deviation equal to zero and ½, respectively. The same white noise has been assigned to the cost of the restoration actions considered. Hence we both considered uncertainty in the benefits and costs of the restoration actions. The truncated noise is included to add further uncertainty which may be related to other factors not considered in the biophysical models and portfolio models: for example species changes in habitat preferences that change their habitat suitability, and changes in the interactions among species which may change the asset correlation. The truncated Gaussian noise is also taking care of all deterministic factors included in the portfolio such as the effectiveness factor, stakeholder preferences, and the extent of military areas.

Thirty Monte Carlo simulations are generated by considering such uncertainties in local values and costs of restoration actions. Thirty simulations are enough to capture the variability of the output based on our previous studies of the uncertainty in the biophysical models [5], [6], [11], [79] by performing a global sensitivity and uncertainty analysis [80].

## Supporting Information

File S1 The file contains supporting materials and methods (Asset Data, Management Area Scale, Biophysical Models and Restoration Actions, Nourishment, and supporting tables: **Table S1,** Restoration actions and their effectiveness for the Snowy Plover (SP). **Table S2,** Restoration actions and their effectiveness for the Piping Plover (PP). **Table S3,** Restoration actions and their effectiveness for the Red Knot (RK). **Table S4,** Restoration actions and their effectiveness for the Military Area (MA). **Table S5,** MCDA model for the Snowy Plover. **Table S6,** MCDA model for the Military Area. **Table S7,** Input factors for the PDM for Santa Rosa Island; Table S8. PDM restoration plan (Pareto set) in 2013 for Santa Rosa Island. **Table S9,** MCDA restoration plan in 2013 for Santa Rosa Island. Supporting figures: **Figure S1,** Local species richness for Santa Rosa Island and occurrences of the three threatened and endangered species considered in the case study. **Figure S2,** Military area of Santa Rosa Island. **Figure S3,** Sea level rise scenario and land cover change. **Figure S4,** Habitat suitability of the Snowy Plover in time for the whole Florida Gulf coast. **Figure S5,** Habitat suitability of the portion of Santa Rosa Island managed by EAFB. **Figure S6,** Frequency distribution of restoration interventions determined by the PDM for selected years in the simulated period (2013–2100).
(PDF)

## References

[1] Aiello-Lammens ME, Chu-Agor ML, Convertino M, Fischer R, Linkov I, et al (2011) The impact of sea level rise on Snowy Plovers in Florida: Integrating hydrological, habitat, and metapopulation models. Global Change Biology 17 (12) 3644–3654.

[2] Convertino M, Kiker GA, Chu-Agor ML, Munoz-Carpena R, Martinez CJ, et al.. (2011) Integrated Modeling to Mitigate Climate Change Risk due to Sea Level Rise of Imperiled Shorebirds on Florida Coastal Military Installations, NATO Book “Climate Change: Global Change and Local Adaptation”, I. Linkov and T. Bridges editors.

[3] Convertino M, Chu-Agor ML, Fischer RA, Kiker GA, Munoz-Carpena R, et al (2012) Coastline Fractality as Fingerprint of Scale-free Shorebird Patch-size Fluctuations due to Climate Change,. Ecological Processes

[4] Convertino M, Elsner J, Kiker GA, Munoz-Carpena R, Martinez CJ, et al (2011) Do Tropical Cyclones Shape Shorebird Patterns? Biogeoclimatology of Snowy Plovers in Florida, PLoS ONE 10.1371/journal.pone.0015683. 10.1371/journal.pone.0015683 PMC3020223 21264268

[5] Chu-Agor ML, Muñoz-Carpena R, Kiker GA, Emanuelsson A, Linkov I (2011) Exploring sea level rise vulnerability of coastal habitats through global sensitivity and uncertainty analysis. Environmental Modelling & Software 26: 593–604.

[6] Chu-Agor ML, Muñoz-Carpena R, Kiker GA, Aiello-Lammens M, Akçakaya R, et al (2012) Simulating the fate of Florida Snowy Plovers with sea level rise: exploring potential population management outcomes with a global uncertainty and sensitivity analysis perspective,. Ecological Modelling

[7] National Academy of Sciences (2012) Ecosystem Services: Charting a Path to Sustainability, Interdisciplinary Research Team Summaries, National Academy of Sciences Press. Available: http://download.nap.edu/cart/download.cgi?&record_id=13331&free=1. Accessed 2012 June 10.

[8] EAFB (2012) Eglin Air Force Base, Florida. 2010–2011 Outdoor Recreation. Hunting and Freshwater Fishing Map. Available: http://www.waltonoutdoors.com/wp-content/uploads/2010/11/RecMap.pdf. Accessed 2012 June 10.

[9] EAFB Plan (2012) Eglin AFB Santa Rosa Island Mission Utilization Plan. US Fish & Wildlife Service, Panama City, FL, 2 December 2005.

[10] Convertino M, Kiker GA, Muñoz-Carpena R, Fischer RA, Linkov I (2011) Scale- and resolution-invariance of suitable geographic range for shorebird metapopulations,. Ecological Complexity doi:10.1016/j.ecocom.2011.07.007

[11] Convertino M, Muñoz-Carpena R, Kiker GA, Chu-Agor ML, Fischer RA, et al (2011a) Epistemic Uncertainty in Predicted Species Distributions: Models and Space-Time Gaps of Biogeographical Data. Ecological Modelling

[12] Convertino M, Donoghue JF, Chu-Agor ML, Kiker GA, Munoz-Carpena R, et al (2011) Anthropogenic Nourishment Feedback on Shorebirds: a Multispecies Bayesian Perspective,. Ecological Engineering

[13] SRIRC, (2012) Available: http://en.wikipedia.org/wiki/Santa_Rosa_Island_Range_Complex. Accessed 2012 June 10.

[14] Linkov I, Satterstrom K, Kiker GA, Bridges T, Benjamin S, et al (2006) From Optimization to Adaptation: Shifting Paradigms in Environmental Management and Their Application to Remedial Decisions. Integrated Environmental Assessment and Management 2: 92–98. 16640324

[15] Linkov I, Cormier S, Gold J, Satterstrom FK, Bridges T (2012) Using our brains to develop better policy. Risk Analysis Volume 32 (Issue 3) 374–380. 10.1111/j.1539-6924.2011.01683.x 22023503

[16] Linkov I., Moberg E. (2012) Multi Criteria Decision Analysis: Environmental Applications and Case Studies, CRC Press.

[17] Greene R, Devillers R, Luther JE, Eddy BG (2011) GIS-Based Multiple-Criteria Decision Analysis,. Geography Compass 10.1111/j.1749-8198.2011.00431.x.

[18] Malczewski J (2006) GIS-based multi-criteria decision analysis: A survey of the literature. International Journal of Geographical Information Science 20 (7) 249–268.

[19] Labiosa WB, Bernknopf R, Hearn P, Hogan D, Strong D, et al.. (2009) The South Florida Ecosystem Portfolio Model; a map-based multi-criteria ecological, economic, and community land-use planning tool: U.S. Geological Survey Scientific Investigations Report 2009–5181, 41 p.

[20] Keisler JM, Sundell RC (1997) Combining Multi-Attribute Utility and Geographic Information for Boundary Decisions:. An Application to Park Planning Journal of Geographic Information and Decision Analysis vol. 1 (no. 2) 100–119.

[21] Andersen MC, Thompson B, Boykin K (2004) Spatial risk assessment across large landscapes with varied land use: lessons from a conservation assessment of military lands. Risk Anal 24 (5) 1231–42. 15563290 10.1111/j.0272-4332.2004.00521.x

[22] Salo A, Keisler J, Morton A (2011) Portfolio Decision Analysis, Improved Methods for Resource Allocation, 1st Edition, XV, 409 p.

[23] Sethi SA (2010) Risk management for fisheries,. Fish And Fisheries 11: 341–365.

[24] Karvetski CW, Lambert JH, Keisler JM, Linkov I (2011) Integration of Decision Analysis and Scenario Planning for Coastal Engineering and Climate Change,. IEEE Transactions On Systems, Man, And Cybernetics-Part A: Systems And Humans VOL. 41 (NO. 1) 63.

[25] Ando AW, Mallory ML (2012) Optimal portfolio design to reduce climate-related conservation uncertainty in the Prairie Pothole Region,. PNAS vol. 109 (no. 17) 6484–6489 doi: 10.1073/pnas.1114653109 22451914 10.1073/pnas.1114653109 PMC3340047

[26] Hoekstra J (2012) Improving biodiversity conservation through modern portfolio theory,. PNAS doi: 10.1073/pnas.1205114109 10.1073/pnas.1205114109 PMC3340081 22517756

[27] Koellner T, Schmitz OJ (2006) Biodiversity, Ecosystem Function, and Investment Risk,. Bioscience 56: 12.

[28] Hills J, Margaret C, Le Tissiera M, Muirc D, Robinson C (2009) Landscape-scale analysis of ecosystem risk and returns: A new tool for ICZM,. Marine Policy Volume 33 (Issue 6) 887–900 Coastal Management in Northwest Europe.

[29] Keisler J, Linkov I (2011) Managing a portfolio of risks. Wiley Encyclopedia of Operations Research and Management Science, Vol. 4: 2960–2983.

[30] Crowe KA, Parker WH (2008) Using portfolio theory to guide reforestation and restoration under climate change scenarios,. Climatic Change 89: 355–370 DOI 10.1007s105840079373x/s10584-007-9373-x

[31] Enrıquez-Andrade RR, Guillermo Vaca-Rodrıguez J (2004) Evaluating ecological tradeoffs in fisheries management: a study case for the yellowfin tuna fishery in the Eastern Pacific Ocean,. Ecological Economics 48: 303–315.

[32] Figge F (2004) Bio-folio: applying portfolio theory to biodiversity. Biodiversity and Conservation 13: 827–849.

[33] Kennedy MC, Ford ED, Singleton P, Finney M, Agee JK (2008) Informed multi-objective decision-making in environmental management using Pareto optimality,. Journal of Applied Ecology 45: 181–192 doi: 10.1111/j.1365-2664.2007.01367.x

[34] Richardson DM, Hellmann JJ, McLachlan JS, Sax DF, Schwartz MW, et al (2009) Multidimensional evaluation of managed relocation,. PNAS vol. 106 (no. 24) 9721–9724 doi: 10.1073/pnas.0902327106 19509337 10.1073/pnas.0902327106 PMC2694035

[35] Moloney PD, Hearne JW, Gordon IJ, Mcleod SR (2010) Portfolio Optimization Techniques For A Mixed-Grazing Scenario For Australia's Rangelands,. Natural Resource Modeling DOI: 10.1111/j.1939-7445.2010.00084.x

[36] Marinoni O, Adkinsb P, Hajkowicz S (2011) Water planning in a changing climate: Joint application of cost utility analysis and modern portfolio theory,. Environmental Modelling & Software Volume 26 (Issue 1) 18–29 Thematic Issue on Science to Improve Regional Environmental Investment Decisions.

[37] Yanmei Q (2001) Applying portfolio theory to the valuation of forest biodiversity. Management Science and Industrial Engineering (MSIE), 2011 International Conference on.

[38] Yemshanov D, Koch FH, Ben-Haim Y, Smith WD (2010) Detection capacity, information gaps and the design of surveillance programs for invasive forest pests,. Journal of Environmental Management 91: 2535e2546. 20674144 10.1016/j.jenvman.2010.07.009

[39] Ziv G, Baran E, Nam S, Rodríguez-Iturbe I, Levin SA (2012) Trading-off fish biodiversity, food security, and hydropower in the Mekong River Basin. Proc Natl Acad Sci USA 109: 5609–5614. 22393001 10.1073/pnas.1201423109 PMC3326487

[40] Kasprzyk JR, Reed PM, Characklis GW, Kirsch BR (2012) Many-objective de Novo water supply portfolio planning under deep uncertainty,. Environmental Modelling & Software 34: 87e104.

[41] Reed P, Kollat JB, Devireddy VK (2007) Using interactive archives in evolutionary multiobjective optimization: A case study for long-term groundwater monitoring design,. Environmental Modelling and Software 22: 5 doi:10.1016/j.envsoft.2005.12.021

[42] Kollat JB, Reed P (2007) A framework for Visually Interactive Decision-making and Design using Evolutionary Multi-objective Optimization (VIDEO),. Environmental Modelling & Software Volume 22 (Issue 12) 1691–1704.

[43] Drechsler M, Frank K, Hanski I, O'Hara RB, Wissel C (2003) Ranking Metapopulation Extinction Risk: from Patterns in Data to Conservation Management,. Ecological Applications 13: 990–998.

[44] Dunkel J, Weber S (2012) Improving risk assessment for biodiversity conservation. PNAS 109 (35) E2304. 22837394 10.1073/pnas.1207485109 PMC3435213

[45] Goldstein JH, Caldarone G, Duarte TK, Ennaanay D, Hannahs N, et al (2012) Integrating ecosystem-service tradeoffs into land-use decisions. PNAS vol. 109 (issue 19) 7565–7570. 22529388 10.1073/pnas.1201040109 PMC3358905

[46] Diwekar U (2005) Green process design, industrial ecology, and sustainability: A systems analysis perspective,. Resources, Conservation and Recycling Volume 44 (Issue 3) 215–235 Sustainability and Renewable Resources—A Special Issue PRES'03–6th Conference on Process Integration, Modelling and Optimisation for Energy Saving and Pollution Reduction.

[47] Xiao N, Bennett DA, Armstrong MP (2007) Interactive evolutionary approaches to multiobjective spatial decision making: A synthetic review, Computers, Environment and Urban Systems, Volume 31, Issue 3, Pages 232–252.

[48] Huang B, Fery P, Xue L, Wang Y (2008) Seeking the Pareto front for multiobjective spatial optimization problems,. International Journal of Geographical Information Science 507–526 DOI:10.1080/13658810701492365

[49] Hildebrandt P, Knoke T (2011) Investment decisions under uncertainty—A methodological review on forest science studies,. Forest Policy and Economics Volume 13 (Issue 1) January 2011 1–15.

[50] Haldane AG, May RM (2011) Systemic risk in banking ecosystems,. Nature 469: 351–355 doi:10.1038/nature09659 21248842 10.1038/nature09659

[51] Roberts SA, Hall GB, Calamai PH (2011) Evolutionary Multi-objective Optimization for landscape system design,. Journal of Geographical Systems Volume 13 (Number 3) 299–326.

[52] Linkov I, Fischer RA, Convertino M, Chu-Agor ML, Kiker GA, et al.. (2011) SERDP Vulnerability Report: Integrated Climate Change and Threatened Bird Population Modeling to Mitigate Operations Risks on Florida Military Installations. Available: http://www.serdp.org/Program-Areas/Resource-Conservation-and-Climate-Change/Natural-Resources/Coastal-and-Estuarine-Ecology-and-Management/RC-1699#factsheet-7371-objective. Accessed 2012 June 10.

[53] Linkov I, Fischer RA, Kiker GA, Munoz-Carpena R, Convertino M, et al (2012) SERDP FINAL REPORT: Integrated Climate Change and Threatened Bird Population Modeling to Mitigate Operations Risks on Florida Military Installations.

[54] Demers SA, Robinson-Nilsen CW (2012) Monitoring Western Snowy Plover Nests with Remote Surveillance Systems in San Francisco Bay, California. Journal of Fish and Wildlife Management Vol. 3 (Issue 1) 123 Available: http://www.fws.gov/arcata/es/birds/WSP/documents/siteReports/California/2012%20Monitoring%20WSP%20nests%20with%20remote%20surveillance%20systems%20in%20San%20Francisco%20Bay.pdf. Accessed 2012 June 10.

[55] Eberhart-Phillips LJ (2012) Population Viability Of Snowy Plovers In Coastal Northern California, MSc Thesis, Humboldt State University.

[56] Koenen M, Utych R, Leslie DM Jr (1996) Methods used to improve Least tern and Snowy Plover nesting success on alkaline flats. Journal of Field Ornithology 67 (2): 281–291.

[57] Lauten DJ, Castelein KA, Farrar JD, Herlyn HG, Kotaich AA (2011) The Distribution and Reproductive Success of the Western Snowy Plover along the Oregon Coast. The Oregon Biodiversity Information Center Institute for Natural Resources Portland State University

[58] Leatherman SP (1989) National Assessment Of Beach Nourishment Requirements- Associated With Accelerated Sea Level Rise, for U.S. EPA Office of Policy, Planning, and Evaluation. Available: http://www.epa.gov/climatechange/effects/downloads/rtc_leatherman_nourishment.pdf. Accessed 2012 June 10.

[59] Leonard LA, Dixon KL, PIlkey OH (1998) A Comparison of Beach Replenishment on the U.S. Atlantic, Pacific, and Gulf Coasts,. Journal of Coastal Research SI #6: 127–140.

[60] Lott CA, Fischer RA (2011) Conservation and Management of Eastern Gulf Coast. Snowy Plovers (Charadrius alexandrinus), ERDC TN-DOER-E28 September 2011.

[61] Pruner R (2010) Assessing Habitat Selection, Reproductive Performance, and The Affects of Anthropogenic Disturbance of the Snowy Plover Along the Florida Gulf Coast. Master's Thesis, University of Florida, Gainesville, USA

[62] Murley JF, Alpert L, Matthews MJ, Bryk C, Woods B, et al.. (2002) Economics Of Florida's Beaches: The Impact Of Beach Restoration, prepared for Florida Department of Environmental Protection Bureau of Beaches and Wetland Resources DEP Contract No. BS014, Final Project Report for Economic Benefits Analysis/Florida Beach Restoration. Available: http://www.dep.state.fl.us/beaches/publications/pdf/phase1.pdf. Accessed 2012 June 10

[63] Hornaday K, Pisani I, Warne B (2007) Recovery plan for the Pacific Coast Population of the Western Snowy Plover, Volume 1: Recovery Plan, Sacramento Fish and Wildlife Office

[64] RAMAS (2011) Available: http://www.ramas.com/. Accessed 2012 June 10.

[65] IUCN (International Union for Conservation of Nature) (2010) Guidelines for using the IUCN Red List categories and criteria: version 8.1. Available: http://intranet.iucn.org/webfiles/doc/SSC/RedList/RedListGuidelines.pdf. Accessed 2011 Sep 6.

[66] Mace GM, Collar NJ, Gaston KJ (2008) Quantification of extinction risk: IUCN's system for classifying threatened species. Conserv Biol 22: 1424–42. 18847444 10.1111/j.1523-1739.2008.01044.x

[67] Akçakaya HR, Mace GM, Gaston KJ, Regan H, Punt A, et al (2011) The SAFE index is not safe. Frontiers in Ecology and the Environment 9: 485–486.

[68] FGDL, Florida Geographic Data Library Documentation (2009) Ffwcc Potential Habitat By Species. Florida Fish and Wildlife Conservation Commission-Fish and Wildlife Research Institute. Available: http://www.fgdl.org/metadata/fgdl_html/pothab_qry_09.htm. Accessed 2012 June 10.

[69] Smith KV (1996) Estimating Economic Values for Nature. Methods for Non-market Valuation. Edward Elgar, Cheltenham, UK.

[70] SLAMM (2011) Sea Level Affecting Marshes Model (SLAMM), Warren Pinnacle, Inc. Available: http://www.warrenpinnacle.com/prof/SLAMM/. Accessed 2012 June 5.

[71] Anderson PSR, Schapire R (2006) Maximum entropy modeling of species geographic distributions. Ecological Modelling 190: 231–259.

[72] Phillips SJ, Miroslav D (2008) Modeling of species distributions with MaxEnt: new extensions and a comprehensive evaluation. Ecography 31: 161–175.

[73] Kim IY, de Weck OL (2004) Adaptive weighted-sum method for bi-objective optimization: Pareto front generation,. Structural and Multidisciplinary Optimization, 01/2005 29 (2) 149–158 DOI:10.1007/s00158-004-0465-1

[74] Mills WL Jr, Hoover WL (1982) Investment in Forest Land: Aspects of Risk and Diversification,. Land Economics Vol. 58 (No. 1) 33–51.

[75] Macmillan WD (1992) Risk and Agricultural Land Use:A Reformulation of the Portfolio-Theoretic Approach to the Analysis of a von Thunen Economy,. Geographical Analysis Vol. 24 (No. 2) Ohio State University Press.

[76] Edwards SF, Link JS, Rountree BP (2004) Portfolio management of wild fish stocks,. Ecological Economics Volume 49 (Issue 3) 317–329 ISSN 0921-8009, 10.1016/j.ecolecon.2004.04.002.

[77] Knoke T, Steinbeis OE, Bösch M, María Román-Cuesta R, Burkhardt T (2011) Cost-effective compensation to avoid carbon emissions from forest loss: An approach to consider price–quantity effects and risk-aversion,. Ecological Economics Volume 70 (Issue 6) 1139–1153 ISSN 0921-8009, 10.1016/j.ecolecon.2011.01.007.

[78] Aerts JCJH, Botzen W, van der Veen A, Krywkow J, Werners S (2008) Dealing with uncertainty in flood management through diversification. Ecology and Society 13 (1) 41.

[79] Convertino M, Chu-Agor ML, Baker K, Linkov I, Munoz-Carpena R (2013) Untangling Drivers of Species Distribution Models: Global Sensitivity and Uncertainty Analysis of MaxEnt,. Environmental Modelling and Software in revision.

[80] Saltelli A, Ratto M, Andres T, Campolongo F, Cariboni J, et al.. (2004) Global Sensitivity Analysis: The Primer, Wiley.

[81] Beale N, Rand DG, Battey H, Croxsond K, Maye RM, Nowak MA (2011) Individual versus systemic risk and the Regulator's Dilemma. PNAS 10.1073/pnas.1105882108 PMC3150885 21768387

[82] Zhou Q, Lambert JH, Karvetski CW, Keisler JM, Linkov I (2012) Flood protection diversification to reduce probabilities of extreme losses. Risk Anal 2012 Nov;32 (11) 1873–87 doi: 10.1111/j.1539-6924.2012.01870.x 10.1111/j.1539-6924.2012.01870.x 22817779

[83] Kennedy MC, Ford DE (2011) Using Multicriteria Analysis of Simulation Models to Understand Complex Biological Systems,. BioScience Vol. 61 (No. 12) 994–1004.

