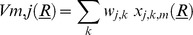# Correction: Portfolio Decision Analysis Framework for Value-Focused Ecosystem Management

**DOI:** 10.1371/journal.pone.0092951

**Published:** 2014-03-18

**Authors:** The PLOS ONE Staff

Equation 1 is incorrect and has been updated.

Please see the corrected Equation 1 here.