# Supplementary material for: Portfolio Decision Analysis Framework for Value-Focused Ecosystem Management
Source: PLoS One. 2013 Jun 18;8(6):e65056. doi: 10.1371/journal.pone.0065056 (PMC3688797; doi:10.1371/journal.pone.0065056)
Supplement: File S1 — The file contains supporting materials and methods (Asset Data, Management Area Scale, Biophysical Models and Restoration Actions, Nourishment, and supporting tables: Table S1, Restoration actions and their effectiveness for the Snowy Plover (SP). Table S2, Restoration actions and their effectiveness for the Piping Plover (PP). Table S3, Restoration actions and their effectiveness for the Red Knot (RK). Table S4, Restoration actions and their effectiveness for the Military Area (MA). Table S5, MCDA model for the Snowy Plover. Table S6, MCDA model for the Military Area. Table S7, Input factors for the PDM for Santa Rosa Island; Table S8. PDM restoration plan (Pareto set) in 2013 for Santa Rosa Island. Table S9, MCDA restoration plan in 2013 for Santa Rosa Island. Supporting figures: Figure S1, Local species richness for Santa Rosa Island and occurrences of the three threatened and endangered species considered in the case study. Figure S2, Military area of Santa Rosa Island. Figure S3, Sea level rise scenario and land cover change. Figure S4, Habitat suitability of the Snowy Plover in time for the whole Florida Gulf coast. Figure S5, Habitat suitability of the portion of Santa Rosa Island managed by EAFB. Figure S6, Frequency distribution of restoration interventions determined by the PDM for selected years in the simulated period (2013–2100). (PDF) [file pone.0065056.s001.pdf]

Supporting Information

Convertino M. (Portfolio Decision Analysis Framework for Value-Focused Ecosystem Management)

SI Materials and Methods

Asset Data

The Snowy Plover, Piping Plover, and Red Knot are the threatened and endangered species (TERs) observed on beach and salt marsh habitats of Florida Gulf coast ecosystem (1-9). Figure S1 shows the occurrences of the abovementioned species for Santa Rosa Island (SRI). SRI is the barrier island, partially managed by EAFB (10-11), that is considered in our study as location where species of concern and intense human use co-occur. The island that is about 30,000 m long and 500 m wide on average and is used for a variety of test and evaluation activities and training exercises by EAFB (10,11). A portion of the military beach area is open for public recreation and it constitutes a suitable habitat for multiple species including the shorebird considered (12). Thus, beach areas support both military activities and species needs. The portion of SRI managed by military is 12.5 km^2 and the portion within the military areas is 2.5 km^2.
Occurrences of these shorebirds for the period 2002-2012 are shown in Figure 1 and Figure S1. Data of the species considered are from the Florida Geographic Data Library (13), and from efforts of previous studies related to the effect of sea level rise on these shorebirds (1,14,15). The data of species from these sources is also integrated from information derived from (16). More information about species, habitat use, and suitability of species is contained in (2-9). Data for the military installations and recreational use are from the Florida Geographic Data Library (13).

The local species richness (LSR), that is the number of unique species occurring in each pixel, is calculated in order to show how SRI is a hotspot of biodiversity and to make evident the importance of considering multiple species for a global conservation of the ecosystem. The pixel size is 120 m^2 that is the unit used in the biophysical modeling. LSR for the Panhandle-Big Bend-Peninsula region is shown in Figure 1. SRI is represented in the inset of Figure 1, and LSR for SRI is shown in Figure S1. The maximum local species richness for SRI is eight and for the whole Florida Gulf coast is ten. The global species richness, that is the total number of unique species in a region, of SRI is sixteen while for the whole Florida Gulf coast is forty-six.

For the human assets, we consider the military beach areas and infrastructure that falls into non-ocean pixels at each year of the analysis. These assets are shown in Figure S2 at the current state. Range operations rely on land-based radar and electro-optical time-space-position-information systems to monitor and transfer test data to the Central Control Facility on Eglin AFB (10-12). These instrumentation systems, located on Santa Rosa Island and other locations provide coverage for test and evaluation activities in the

Gulf of Mexico (12). The use of SRI is evolving due to changes in threats to national security and the effects of hurricanes over the past decade. The SRI complex is used for Expanded Surf Zone Testing/Training, Mine Clearing Testing, Beach Obstacle Clearing and Neutralization, Small Boat Obscurant Testing, Live Fire, Expanded Special Operations Training, Amphibious Assaults, and other types of activities.

Management Area Scale

The extent of management areas is calculated by evaluating the tradeoff between the spatial needs for a correct representation of the assets (species and military areas) and a feasible scale for the implementation of restoration actions.
The average home range of species, that is the average distance at which a species lives and disperses in the ecosystem, is a good indicator for the minimal dimension of the management area. The scale of infrastructure is in our case larger than the scale of the species considered. We consider the average home range of the Snowy Plover (120 m) that is the ``sentinel species'' of the beach habitat in the Florida Gulf coast (17), and the average shore length of the nourishment (7620 m) (9,18,19) that is the largest restoration action considered in this study.
Thus, the average scale of a management area is 3750 m that is the average of the two abovementioned distances. For simplicity, the management area is assumed to be a square. Hence, each management area contains about 961 pixels used in the biophysical modeling. Values of spatially explicit criteria from biophysical models are averaged within each management area to obtain values of assets as a function of restoration actions at the appropriate scale for the portfolio decision model (PDM).

Biophysical Models and Restoration Actions

A land cover model (SLAMM) (19), a habitat suitability model (MaxEnt) (20-21), and a metapopulation model (RAMAS) (22) are used to reproduce the evolution of habitat area and quality, and the risk of assets as a function of sea level rise and restoration plans from 2013 to 2100 (1,2-9). Biophysical models are run at the resolution of 120 m^2.
A complete explanation of these models and their application to the scenario with no restoration action is included in (1) and (3). In this paper we rerun this set of models for the nourishment scenarios selected by the MCDA and the PDM. Models are run at the scale of the whole Gulf coast of Florida. However, MCDA and PDM consider only Santa Rosa Island that is managed by EAFB. Thus, the variability of restoration actions is considered only at the scale of Santa Rosa Island and no action is assumed elsewhere. Despite we focus only at the scale of installation management restoration plans chosen at SRI influence habitat and metapopulation dynamics outside SRI.
The outputs of biophysical models are the inputs of the MCDA model (Tables S4 and S5) as explained in Materials and Methods.

Nourishment

In order to simulate the nourishment scenarios at SRI, we adopt the SLAMM model modified by (24). We consider the A1B sea level rise scenario rescaled to 2 m in 2100. The modified version of SLAMM takes into account time-specific changes in the land

cover that are caused by the nourishment. These changes are simulated by adding another term to the equation that describes the evolution of the elevation *z* of pixels used in the biophysical modeling. The equation considers the natural accretion/sedimentation rate *a* in the time period Δt (where Δt=1 year) sea level rise SLR, and the increase in elevation Δz dictated by the nourishment, as follows

$$z(t) = z(t-1) + \Delta t\, a - SLR(t) + \Delta z(t) \quad \text{[S1]}$$

By using Eq. S1, the modified SLAMM considers changes in elevation, slope and land cover classes at a specific year as a function of both natural and anthropogenic drivers. If beach nourishment is carried out in any management area SLAMM increases *z(t)* of beach cells of Δz = 1 m regardless of the location and the year considered. However, heterogeneous changes in space and time due to nourishment can be implemented.

References

1. Aiello-Lammens, M.E., Chu-Agor, M. L., Convertino, M., Fischer, R., Linkov, I., and Akçakaya, H.R., (2011) The impact of sea level rise on Snowy Plovers in Florida: Integrating hydrological, habitat, and metapopulation models. Global Change Biology 17(12): 3644-3654.

2. Convertino, M., G.A. Kiker, M.L. Chu-Agor, R. Munoz-Carpena, C.J. Martinez, M. Aiello-Lammens, R.H. Akcakaya, R.A. Fischer, I. inkov, (2011) Integrated Modeling to Mitigate Climate Change Risk due to Sea Level Rise of Imperiled Shorebirds on Florida Coastal Military Installations, NATO Book ``Climate Change: Global Change and Local Adaptation'', I. Linkov and T. Bridges editors

3. Convertino, M., M.L. Chu-Agor, R.A. Fischer, G. Kiker, R. Munoz-Carpena, I. Linkov (2012), Coastline Fractality as Fingerprint of Scale-free Shorebird Patch-size Fluctuations due to Climate Change, Ecological Processes.

4. Convertino, M., J. Elsner, G. Kiker, R. Munoz-Carpena, Martinez, C.J., R. Fischer, I. Linkov (2011), Do Tropical Cyclones Shape Shorebird Patterns? Biogeoclimatology of Snowy Plovers in Florida, PLoS ONE, 10.1371/journal.pone.0015683

5. Chu-Agor, M.L. , R Muñoz-Carpena, G. Kiker, A. Emanuelsson, and I. Linkov, (2011) Exploring sea level rise vulnerability of coastal habitats through global sensitivity and uncertainty analysis. Environmental Modelling & Software 26:593-604.

6. Chu-Agor, M.L., R. Muñoz-Carpena, G.A. Kiker, M. Aiello-Lammens, R. Akçakaya, M. Convertino, I. Linkov, (2011) Simulating the fate of Florida Snowy Plovers with sea level rise: exploring potential population management outcomes with a global uncertainty and sensitivity analysis perspective, Ecological Modelling

7. Convertino, M., G. Kiker, R. Muñoz-Carpena, R. Fischer, I. Linkov. (2011) Scale- and resolution-invariance of suitable geographic range for shorebird metapopulations, Ecological Complexity, doi:10.1016/j.ecocom.2011.07.007

8. Convertino, M., Muñoz-Carpena, R.,, Kiker, G.A., Chu-Agor, M., Fischer, R. and Linkov, I. (2011) Epistemic Uncertainty in Predicted Species Distributions: Models and Space-Time Gaps of Biogeographical Data. Ecological Modelling

9. Convertino, M., Donoghue, J.F., Chu-Agor, M., M., Kiker, G.A., Munoz-Carpena, R., Fischer, R. and Linkov, I. (2011) Anthropogenic Nourishment Feedback on Shorebirds: a Multispecies Bayesian Perspective, Ecological Engineering.

10. EAFB, (2012) Eglin Air Force Base, Florida. 2010 - 2011 Outdoor Recreation,. Hunting and Freshwater Fishing Map, http://www.waltonoutdoors.com/wp-content/uploads/2010/11/RecMap.pdf

11. EAFB Plan, (2012) Eglin AFB Santa Rosa Island Mission Utilization Plan. US Fish & Wildlife Service, Panama City, FL, 1 December 2005

12. SRIRC, (2012) http://en.wikipedia.org/wiki/Santa_Rosa_Island_Range_Complex

13. FGDL, Florida Geographic Data Library Documentation, Title: Ffwcc Potential Habitat By Species, (2009) Florida Fish and Wildlife Conservation Commission-Fish and Wildlife Research Institute, http://www.fgdl.org/metadata/fgdl_html/pothab_qry_09.htm

14. Lott C.A. and Richard A. Fischer, (2011) Conservation and Management of Eastern Gulf Coast. Snowy Plovers (Charadrius alexandrinus), ERDC TN-DOER-E28 September 2011

15. Pruner R., (2010) Assessing Habitat Selection, Reproductive Performance, and The Affects of Anthropogenic Disturbance of the Snowy Plover Along the Florida Gulf Coast. Master's Thesis, University of Florida, Gainesville, USA

16. Endries M., Beth Stys, Gary Mohr, Georgia Kratimenos, Susan Langley, Karen Root, Randy Kautz, 2000, Wildlife Habitat Conservation Needs in Florida Updated Recommendations for Strategic Habitat Conservation Areas, FISH AND WILDLIFE RESEARCH INSTITUTE, ISSN 1930-1448, http://myfwc.com/media/1205682/TR15.pdf

17. Linkov Igor, Richard A. Fischer, Gregory A. Kiker, Rafael Munoz-Carpena, Matteo Convertino, Ma. Librada Chu-Agor, Anna Linhoss, Matthew Aiello-Lammens, Christopher Martinez, Resit Akçakaya, (2012) SERDP FINAL REPORT: Integrated Climate Change and Threatened Bird Population Modeling to Mitigate Operations Risks on Florida Military Installations

18. Linkov I., Richard A. Fischer, Matteo Convertino, Ma. Librada Chu-Agor, Gregory A. Kiker Rafael Munoz-Carpena, Christopher Martinez, Resit Akçakaya, Matthew Aiello-Lammens (2011) SERDP Vulnerability Report: Integrated Climate Change and Threatened Bird Population Modeling to Mitigate Operations Risks on Florida Military Installations, http://www.serdp.org/Program-Areas/Resource-Conservation-and-Climate-Change/Natural-Resources/Coastal-and-Estuarine-Ecology-and-Management/RC-1699#factsheet-7371-objective

19. Leatherman S.P., (1989) National Assessment Of Beach Nourishment Requirements-Associated With Accelerated Sea Level Rise, for U.S. EPA Office of Policy, Planning, and Evaluation, http://www.epa.gov/climatechange/effects/downloads/rtc_leatherman_nourishment.pdf

20. SLAMM (2011) Sea Level Affecting Marshes Model (SLAMM), Warren Pinnacle, Inc. (http://www.warrenpinnacle.com/prof/SLAMM/).

21. Phillips S, Anderson R, Schapire R., (2006) Maximum entropy modeling of species geographic distributions. Ecological Modelling 190: 231–259.

22. Phillips SJ, Miroslav D., (2008) Modeling of species distributions with MaxEnt: new extensions and a comprehensive evaluation. Ecography 31: 161–175.

23. RAMAS, (2011) http://www.ramas.com/

24. Chu-Agor, M.L., Guzman, J.A. Muñoz-Carpena, R., Kiker, G.A., Linkov, I., (2012) Changes in beach habitat due to the combined effects of long-term sea level rise, storm erosion, and nourishment, Environmental Modelling and Software

Snowy Plover	
**Restoration Actions**	**Effectiveness Factor**
Restore Ephemeral Pools/Beach Profile	0.9
Restore Dune Vegetation	0.8
Monitor	0.5
Nourishment	1
Predator Management	0.6
Limitation Recreational Use	0.5
No Action	1

Table S1. Restoration actions and their effectiveness for the Snowy Plover (SP). Each restoration action is characterized by an effectiveness factor that is proportional to the probability of success of each restoration action for each asset locally. The effectiveness factor is assessed after expert judgement and review of literature. The effectiveness is assumed equal to one when the effects of restoration actions are predicted by biophysical models. This is because the criteria values in the MCDA model consider already the effects of the simulated restoration action. In our case the baseline scenario (no restoration actions) is modeled in (1), and in (3). The nourishment is modeled as in (18) and in (24).

Piping Plover	
**Restoration Actions**	**Effectiveness Factor**
Restore Ephemeral Pools/Beach Profile	0.8
Restore Dune Vegetation	0.4
Monitor	0.5
Nourishment	1
No Action	1

Table S2. Restoration actions and their effectiveness for the Piping Plover (PP). The effectiveness is assumed equal to one when the effects of restoration actions are predicted by biophysical models.

Red Knot	
**Restoration Actions**	**Effectiveness Factor**
Restore Ephemeral Pools/Beach Profile	0.6
Monitor	0.3
Nourishment	1
No Action	1

Table S3. Restoration actions and their effectiveness for the Red Knot (RK). The effectiveness is assumed equal to one when the effects of restoration actions are predicted by biophysical models.

Military Area	
**Restoration Actions**	**Effectiveness Factor**
Nourishment	1
No Action	1

Table S4. Restoration actions and their effectiveness for the Military Area (MA). The effectiveness is assumed equal to one when the effects of restoration actions are predicted by biophysical models.

Criteria			Sub-Criteria				*Assets*	Snowy Plover 1	Snowy Plover 1
**Name**	**Weight**	**Normalized Weight**	**Name**	**Maximize(1) / Minimize(0)**	**Weight**	**Normalized Weight**	***Restoration Actions***	No Action	Nourishment
Subpopulation Viability	2	0.600	Abundance	1	3	0.600		50	40
			Abundance Fluctuation	0	2	0.400		0.5	0.8
Habitat Quality	1	0.400	Habitat Area	1	3	0.273		20	40
			Habitat Suitability	1	4	0.364		0.7	0.9
			Fragmentation (D_f)	0	2	0.182		0.8	0.3
			Patch Connectivity	1	2	0.182		0.5	0.85

Table S5. MCDA model for the Snowy Plover. Criteria at different order characterize each restoration action for the SP in all the management areas of SRI. Thus, criteria values of the MCDA model vary in each management area for the same action considered. The weights are assigned by expert judgement. In our case study we run the whole set of models (SLAMM, MaxEnt, and RAMAS) (20-23) for the no-action and the nourishment (1,24). We calculate the MCDA value of other restoration actions by multiplying the effectiveness factor of each restoration action to the MCDA value of the no-action (1). For the PP and the RK the subpopulation viability criteria are calculated using the statistical method of (3) rather than using RAMAS. The abundance of SP and RK is calibrated on abundance data in the suitable patches and rescaled to the predicted patches proportionally to their area for every year simulated. The weight of criteria for the PP and RK are the same of the weights for the SP.

Criteria			Sub-Criteria			*Assets*	Military Area 1	Military Area 1
**Name**	**Weight**	**Weight (Norm.)**	**Name**	**Maximize(1) / Minimize(0)**	**Weight**	***Restoration Actions***	No Action	Nourishment
Mission Success	1	0.500	Infrastructure & Mission Test Site Area	1	2		5	10
Training Area Quality	1	0.500	Training Area	1	2		10	20
			Training Suitability	1	1		1	1

Table S6. MCDA model for the Military Area. Criteria at different order characterize each restoration action for the military area in all the management areas of SRI. Thus, criteria values of the MCDA model vary in each management area for the same action considered. Infrastructure and mission test sites are represented in Figure S2 within dotted and continuous lines (from A1 to A18). Training areas are the pink areas in Figure S2 that are closed to all forms of public access. We assume that their training suitability is the same and equal to 1 in a range [0, 1]. In our case study we run the whole set of models (SLAMM, MaxEnt, and RAMAS) (20-23) for the no-action and the nourishment (1-24).

Asset	Management Areas	Restoration Actions	Cost
Military Area	1,2,3,4,5,6,7,8	Nourishment	50
	1,2,3,4,5,6,7,8	No Action	1
Piping Plover	2,3,4,5,6,7,8	Restore Ephemeral Pools/Beach Profile	10
	2,3,4,5,6,7,8	Restore Salt Marsh	80
	2,3,4,5,6,7,8	Monitor	5
	2,3,4,5,6,7,8	Nourishment	50
	2,3,4,5,6,7,8	No Action	0
Snowy Plover	1,2,3,4,5,6,7,8	Restore Ephemeral Pools/Beach Profile	10
	1,2,3,4,5,6,7,8	Restore Dune Vegetation	15
	1,2,3,4,5,6,7,8	Monitor	5
	1,2,3,4,5,6,7,8	Nourishment	50
	1,2,3,4,5,6,7,8	Predator Management	25
	1,2,3,4,5,6,7,8	Limitation Recreational Use	15
	1,2,3,4,5,6,7,8	No Action	0
Red Knot	1	Restore Ephemeral Pools/Beach Profile	10
	1	Monitor	5
	1	Nourishment	50
	1	No Action	0

Table S7. Input factors for the PDM for Santa Rosa Island. A number is assigned to each management area in which each asset occurs. Restoration actions are asset-specific or they benefit multiple assets at the same time in the same management area and in adjacent management areas. The value of each restoration action is the expected local value from the MCDA model and adjusted by the asset vulnerability that considers the whole restoration plan, and the effectiveness factor of each restoration action that considers the local effect of each action. The cost is the cost of each action at the management area scale. We considered costs derived from the literature available and from experts.

Restoration Plan PDM 2013				
Management Area	Asset	Restoration Intervention	Expected Local Value	Cost
1	All	No Action	0.000	0
2	All	No Action	0.000	0
3	Piping Plover	Monitor	0.001	5
3	Snowy Plover (PP)	Restore Ephemeral Pools/Beach Profile	0.027	10
4	Snowy Plover (PP)	Restore Ephemeral Pools/Beach Profile	0.043	10
4	Piping Plover	Monitor	0.001	5
5	Snowy Plover (PP)	Restore Ephemeral Pools/Beach Profile	0.068	10
5	Military Area (SP, PP)	Nourishment	0.060	50
5	Piping Plover	Monitor	0.001	5
6	Snowy Plover (PP)	Restore Ephemeral Pools/Beach Profile	0.082	10
6	Military Area (SP, PP)	Nourishment	0.181	50
6	Piping Plover	Monitor	0.001	5
7	Snowy Plover (PP)	Restore Ephemeral Pools/Beach Profile	0.070	10
7	Military Area (SP, PP)	Nourishment	0.150	50
7	Piping Plover	Monitor	0.001	5
8	Piping Plover (SP)	Restore Ephemeral Pools/Beach Profile	0.015	10
8	Snowy Plover	Restore Dune Vegetation	0.020	15

Table S8. PDM restoration plan (Pareto set) in 2013 for Santa Rosa Island. The set is visualized in Figure 5b. Each Pareto set corresponds to a management plan with a global value $V_T(\underline{R})$ (Eq. 2) at the installation scale. The value in the table is the expected local value of each restoration intervention that is the restoration action selected by the Pareto optimization among all the possible actions that are part of possible restoration plans in the PDM. This value considers also the value of other assets that benefit from the same action selected for other assets. The restoration interventions in common to the MCDA-based plan are evidenced in red. The assets that benefit from the restoration intervention selected for other assets are shown in a lighter tone within parenthesis after the asset for which the intervention is selected.

Restoration Plan MCDA 2013				
**Management Area**	**Asset**	**Restoration Intervention**	**Expected Local Value**	**Cost**
1	Snowy Plover (PP, MA, RK)	Nourishment	0.075	50
2	All	No Action	0.000	0
3	Snowy Plover (PP, MA)	Nourishment	0.113	50
4	All	No Action	0.000	0
5	All	No Action	0.000	0
6	Military Area (SP, PP)	Nourishment	0.190	50
7	Military Area (SP, PP)	Nourishment	0.152	50
8	Military Area (SP, PP)	Nourishment	0.110	50

Table S9. MCDA restoration plan in 2013 for Santa Rosa Island. The set is visualized in Figure 5b. Each set corresponds to a management plan with a global value $V_T(\underline{R})$ (Eq. 2). The value in the table is the local value of each restoration intervention that is the restoration action with the maximum MCDA value among all the possible actions that are part of feasible restoration plans. This value considers also the value of other assets that benefit from the same action selected for other assets. The restoration interventions in common to the PDM-based plan are evidenced in red. The assets that benefit from the restoration intervention selected for other assets are shown in a lighter tone within parenthesis after the asset for which the intervention is selected.

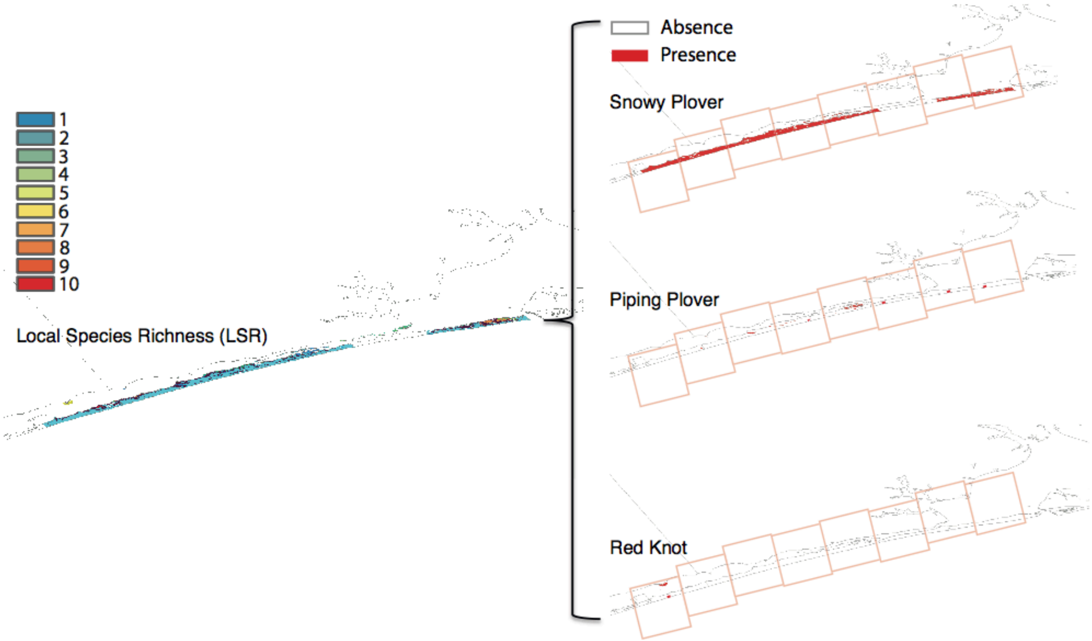

Figure S1. Local species richness for Santa Rosa Island and occurrences of the three threatened and endangered species considered in the case study. The occurrences of Snowy Plover, Piping Plover, and Red Knot, which are shown in red in the right plots (each pixel of 120 m^2 is marked in red if at least one species occurrence is detected), are the input of MaxEnt that is the habitat suitability model used in this study (7).

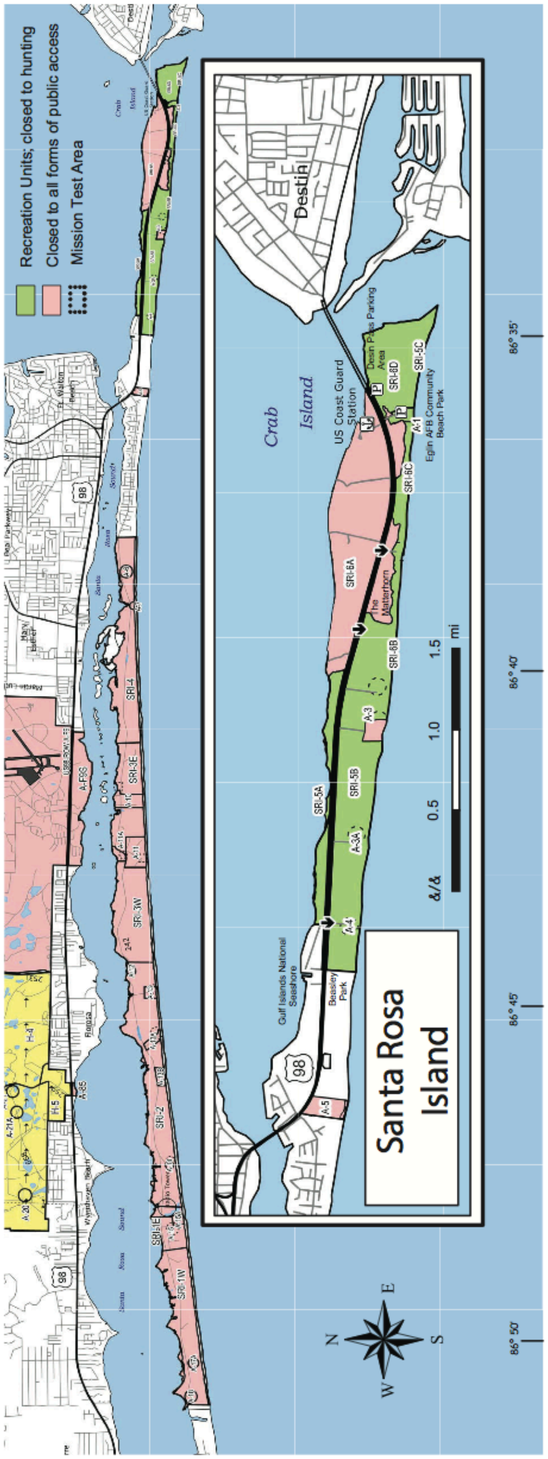

Figure S2. Military area of Santa Rosa Island. The military area is composed by mission test areas that are mostly occupied by infrastructure or areas with intense training, areas that are closed to all form of public access, and recreational areas that are managed by EAFB but open to the public (10-12). We consider only the portion of Santa Rosa Island that is managed by Eglin Air Force Base and the area in the immediate vicinity (white portion of SRI in the figure).

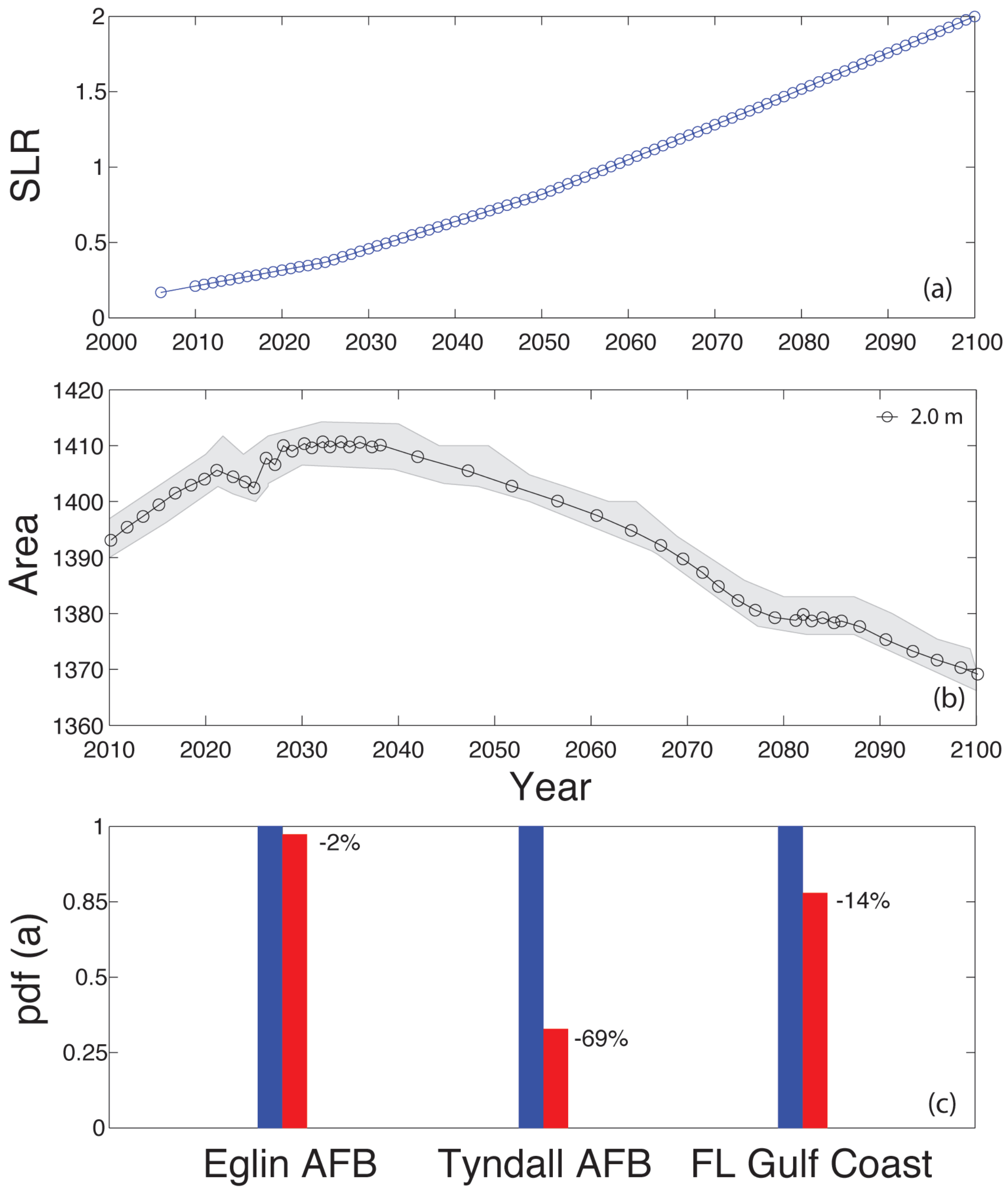

Figure S3. Sea level rise scenario and land cover change. (a) A1-B sea level rise scenario rescaled to 2 m. (b) Change of habitat area consisting in estuarine beach, tidal flat, and ocean beach for Santa Rosa Island. (c) Comparison of the changes in habitat area for Eglin Air Force Base, Tyndall Air Force Base, and the whole Gulf coast of Florida (Figure 1) between 2013 and 2100 for the 2 m sea level rise scenario. Numbers on top of the bars represent the percentage of change in habitat area between 2013 and 2100.

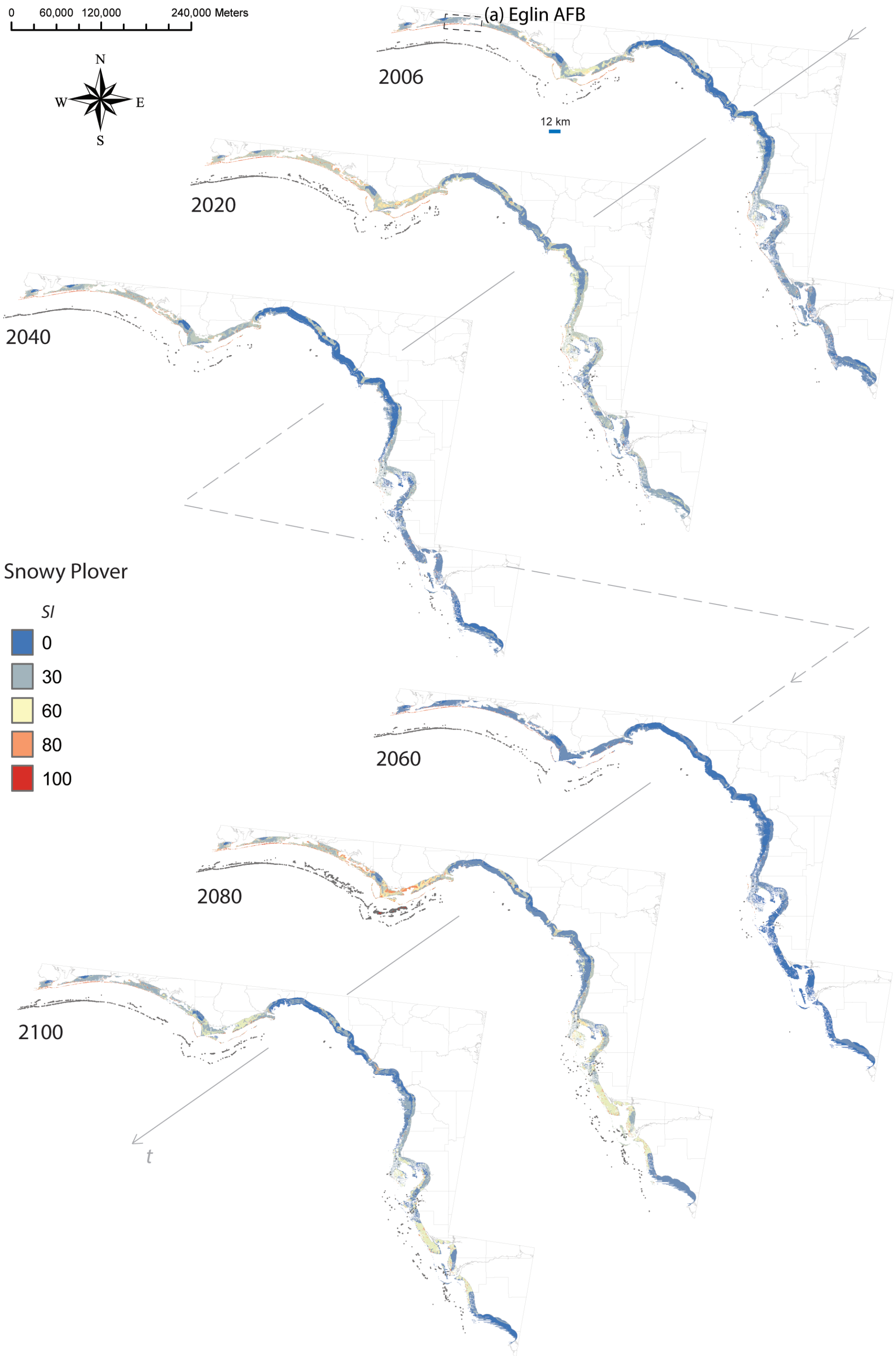

Figure S4. Habitat suitability of the Snowy Plover in time for the whole Florida Gulf coast. The patches used in the metapopulation model (1,3) are represented in grey for each year. SI is the suitability index. The patches are defined where SI>60 and where a minimum population area is guaranteed (3). For clarity of representation the patches are shown detached from the coastline.

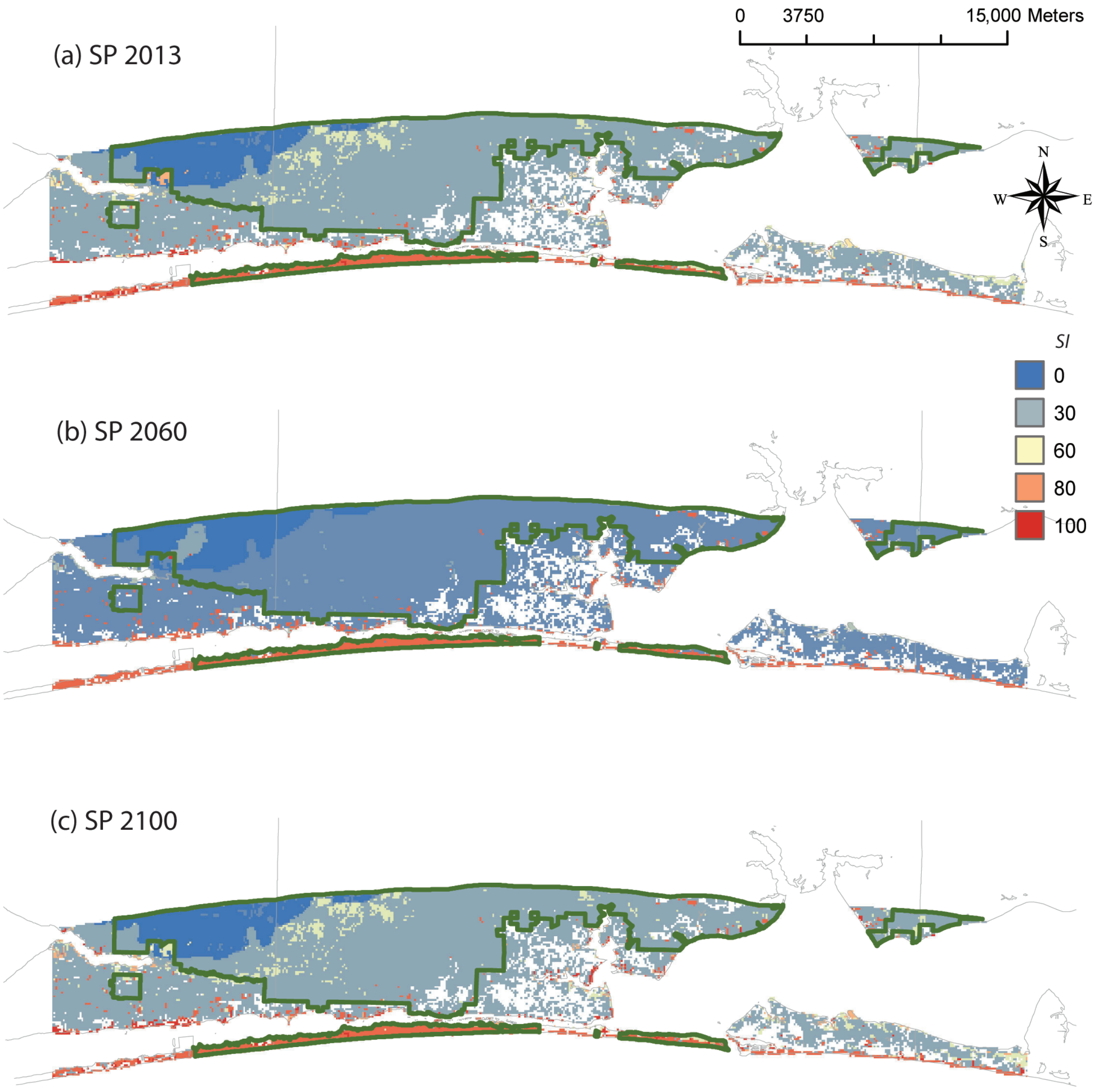

Figure S5. Habitat suitability of the portion of Santa Rosa Island managed by EAFB. The maps are produced by zooming Figure S4 on SRI.

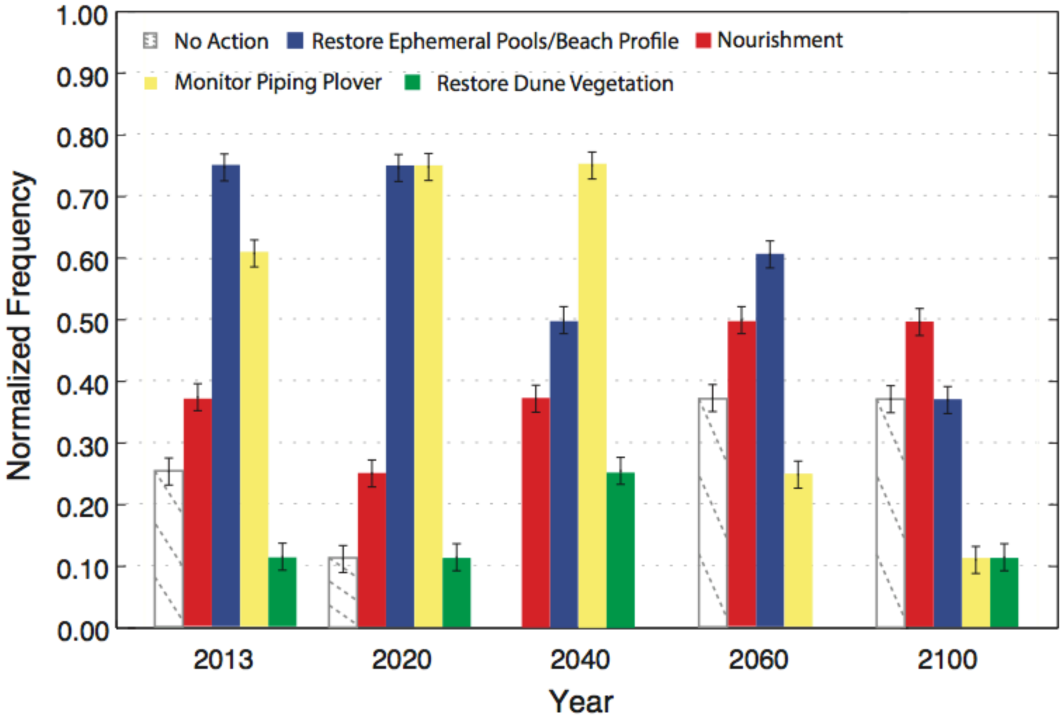

Figure S6. Frequency distribution of restoration interventions determined by the PDM for selected years in the simulated period (2013 - 2100). The frequency of interventions is presented for the Santa Rosa Island managed by EAFB. Error bars are calculated on 30 Monte Carlo simulations performed by perturbing the expected local values of restoration actions with a white noise in a range [-0.05, 0.05]
